# Development of Tailored Graphene Nanoparticles: Preparation, Sorting and Structure Assessment by Complementary Techniques

**DOI:** 10.3390/molecules28020565

**Published:** 2023-01-05

**Authors:** Kaiyue Hu, Luigi Brambilla, Patrizia Sartori, Claudia Moscheni, Cristiana Perrotta, Lucia Zema, Chiara Bertarelli, Chiara Castiglioni

**Affiliations:** 1 Dipartimento di Chimica, Materiali e Ingegneria Chimica Giulio Natta, Politecnico di Milano, 20133 Milano, Italy; 2 Dipartimento di Scienze Biomediche per la Salute, Università degli Studi di Milano, 20133 Milano, Italy; 3 Dipartimento di Scienze Biomediche e Cliniche, Università degli Studi di Milano, 20157 Milano, Italy; 4 Sezione di Tecnologia e Legislazione Farmaceutiche “M. E. Sangalli”, Dipartimento di Scienze Farmaceutiche, Università degli Studi di Milano, 20133 Milano, Italy; 5 Center for Nano Science and Technology@PoliMi, Istituto Italiano di Tecnologia, 20133 Milano, Italy

**Keywords:** graphene-nanoparticles, drug delivery platform, Raman spectroscopy, infrared spectroscopy, transmission electron microscopy, chemical functionalization

## Abstract

We present a thorough structural characterization of Graphene Nano Particles (GNPs) prepared by means of physical procedures, i.e., ball milling and ultra-sonication of high-purity synthetic graphite. UV-vis absorption/extinction spectroscopy, Dynamic Light Scattering, Transmission Electron Microscopy, IR and Raman spectroscopies were performed. Particles with small size were obtained, with an average lateral size <L> = 70–120 nm, formed by few <N> = 1–10 stacked layers, and with a small number of carboxylic groups on the edges. GNPs relatively more functionalized were separated by centrifugation, which formed stable water dispersions without the need for any surfactant. A critical reading and unified interpretation of a wide set of spectroscopic data was provided, which demonstrated the potential of Specular Reflectance Infrared Spectroscopy for the diagnosis and quantification of chemical functionalization of GNPs. Raman parameters commonly adopted for the characterization of graphitic materials do not always follow a monotonic trend, e.g., with the particle size and shape, thus unveiling some limitations of the available spectroscopic metrics. This issue was overcome thanks to a comparative spectra analysis, including spectra deconvolution by means of curve fitting procedures, experiments on reference materials and the exploitation of complementary characterization techniques.

## 1. Introduction

The extremely interesting properties of graphene, such as the high mechanical strength, great electrical [1,2] thermal [3] and magnetic properties [4], and unique optical properties, have made it a potential candidate to be used in several fields of material science and technology [5,6,7,8,9]. Moreover, the acceptable biocompatibility, the very large surface areas [10,11], together with facile functionalization of graphene-based nanoparticles (GNPs) and of graphene derivatives such as graphene oxide (GO) and reduced graphene oxide (rGO) [12,13] have engrossed several researchers in the pharmaceutical and bio-medical fields for applications such as drug/gene delivery systems and tissue engineering [14,15,16,17]. GNPs suitable for the development of drug delivery platforms are required to have an average lateral size <L> in the range of 10–10^2^ nm and an average thickness <N> of a few (1–10) stacked layers [14].

GNPs are systems intrinsically characterized by delocalized π electrons; however, the description of their electronic structure, vibrational dynamics and spectroscopic response cannot be performed under the hypothesis of infinitely extended graphene sheets (ideal 2D crystal), because of the confinement [18,19,20,21] (finite particles size) and of the structure of the edges [21,22], which affect their electronic structure and the spectroscopic response. On the other hand, GNP samples usually are a heterogeneous collection of particles containing a variety of individual objects rather than well-defined and monodisperse molecular species, which, depending on the method of preparation, can differ in size, shape and relative concentration, showing edges with different structures and possibly chemical or structural defects both on the borders and on the graphene basal planes [23,24,25]. The samples containing graphene particles are characterized by a less or more sharp size distribution peaking between the diameter of a large molecule (1–10 nm) [26] and that of a crystal (i.e., extended graphene sheet or micro-crystalline graphite).

As a consequence, GNPs spectra can simultaneously show features typical of disordered solid-state crystalline graphite [18,27,28] as well as those typical of large Polycyclic Aromatic Hydrocarbons (PAHs) [26,29]. The appearance of crystal features vs. molecular features is mostly ruled by the size of the particles and by the structural/chemical defects of the edges [30]. Indeed, for a correct interpretation of the spectroscopic signals of the GNPs, we need to highlight whether we are approaching the molecular or the solid-state regime. For the above reasons, it is challenging to find empirical spectroscopic parameters that allow for a reliable classification of nanosized graphene/graphite particles according to their most relevant structural characteristics. To this aim, C. Backes et al. [31,32] developed “spectroscopic metrics” for the determination of <L> and <N> of liquid-exfoliated few-layer graphene nanosheets. However, it is apparent that a “metric” is often valid for GNPs sharing a set of common characteristics and properties, and it cannot be applied straightforwardly to GNPs samples of different origins or methods of preparation.

In this paper, we present a detailed structural study of GNPs samples we prepared starting from high-purity synthetic graphite by means of simple physical procedures, namely ball milling followed by sonication and centrifugation (details in Section 3). In Table 1, we list the samples obtained at the different preparation steps and their acronyms. 

**Figure 1:**
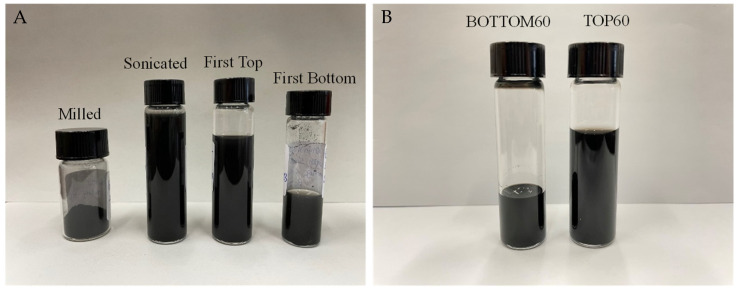
GNP samples, at the different steps of production: (**A**) Nanoparticles samples after milling, sonication, and first centrifugation steps (First Top and First Bottom). (**B**) Nanoparticles after second centrifugation (TOP60 and BOTTOM60). BOTTOM60 and TOP60 dispersion are stable in water.

The method allows for obtaining GNPs that show different lateral sizes and thicknesses and different degrees of functionalization (Figure 1A,B). In the paper, we focus on two specific GNP samples, hereafter referred to as TOP60 and BOTTOM60, respectively, which form stable dispersions in water. The spectroscopy and microscopy analyses show that these GNPs have lateral sizes suitable to make them potential candidates for applications as drug carriers and are formed by a few stacked layers. The large surface area of TOP60 and BOTTOM60 GNPs is expected to favor the uploading of drugs, e.g., through π-π interaction. Moreover, BOTTOM60 samples show the presence of functional groups on the edges of the graphene layers, useful for a subsequent functionalization aiming at the improvement of the stability in a biological environment, as well as the targeting capability.

We characterized the samples by means of several spectroscopic techniques, namely UV-vis absorption spectroscopy (UV-vis), infrared spectroscopy (IR) in Double Transmission (DT) and Specular Reflection (SR) [33,34,35], and multi-wavelength Raman spectroscopy on solid samples deposited on a substrate, combined to dynamic light scattering (DLS) in water dispersion and transmission electrons microscopy (TEM) images of dried samples. Organized as a case study, the paper highlights the subtle interplay of GNPs’ structural characteristics, such as the size, the thickness, the degree of order on the basal plane and on the edges, and the chemical functionalization [36,37], in determining their spectroscopic response. Eventually, the combined methods allow the prediction of the main characteristics of GNPs obtained from any other source.

## 2. Results and Discussion

### 2.1. GNPs Size Distribution by DLS

**Figure 2:**
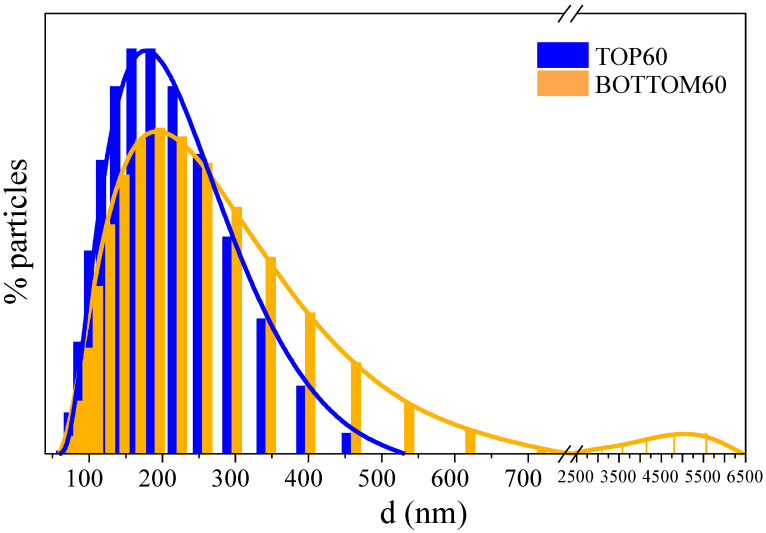
Dynamic light scattering (DLS) diameter (d) distribution curve of TOP60 and BOTTOM60.

The Dynamic Light Scattering (DLS) experiment measures the hydrodynamic diameters (d) of a distribution of ideally spherical particles, which should give rise to the same pattern of light intensity fluctuation as that one obtained from the sample under investigation [38,39]. In this way, the DLS data provide an estimation of the characteristic GNPs dimensions and information on their aggregation state [40,41]. The average d values and sizes distributions of the GNPs and GNPs aggregates obtained are shown in Supplementary Materials Table S1 and Figure 2, respectively. There are some differences between the TOP60 and BOTTOM60 samples; TOP60 exhibits a sharper diameter distribution with a peak located at 177 ± 2 nm, while BOTTOM60 shows a wider distribution with a peak at 190 ± 5 nm. Moreover, in the BOTTOM60 sample, we noticed the presence of some big aggregates with very large hydrodynamic diameters of a few micrometers (see Figure 2). The presence of such large GNPs aggregates suggests that the BOTTOM60 GNPs are more prone to develop inter-particle interactions than the TOP60. This peculiarity can also explain why, after some weeks from the preparation of the water dispersion of BOTTOM60, we observe with the naked eye black aggregates floating in the water while the aqueous dispersion of TOP60 remains clear. This evidence suggests that TOP60 and BOTTOM60 may possess a different number of functional groups, which can promote the aggregation of GNPs. The following discussion, based on the IR and UV-vis spectra analysis, supports this hypothesis. 

As it will be better clarified after TEM analysis, the average d values from DLS must be carefully considered because of two main issues:

(a) In the case of isolated, thin GNPs floating in water, the aspect ratio of the individual layers of nanoparticles is high, so the “equivalent” sphere model cannot capture the right average size.
(b) Graphene nanoparticles are hydrophobic and can form small clusters in water. For this reason, the estimated average diameter of the particles also takes into account the presence of clusters containing a few GNPs. Since the water dispersion is very stable, especially in the case of TOP60 GNPs, these clusters, if present, are small; namely, they do not reach the critical size for precipitation.

Considering point (b), we can argue that the average DLS diameter overestimates the average size of the individual GNPs since the clusters cannot be smaller than a single particle. On the other hand, DLS allows distinguishing between the TOP and BOTTOM nanoparticles, showing that the BOTTOM60 contains a higher concentration of larger aggregates.

### 2.2. GNPs Size from TEM Images

**Figure 3:**
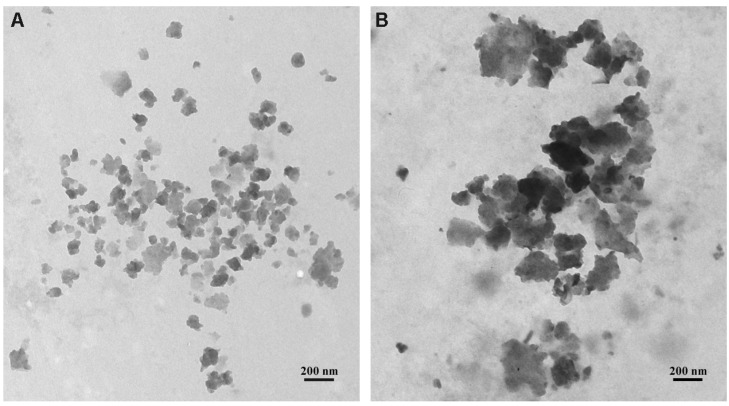
Representative transmission electron microscopy images of (**A**) TOP60 and (**B**) BOTTOM60 GNPs. Both nanoparticles show a similar irregular morphology while exhibiting a different lateral size. TOP60 GNPs have an average lateral dimension of 70 nm, while BOTTOM60 GNPs of 120 nm. Furthermore, BOTTOM60 nanoparticles exhibit a pronounced tendency to entangle with each other in a random manner, producing multilayer aggregates more frequently than TOP60 ones.

Figure 3 shows representative TEM images of TOP60 (A) and BOTTOM60 (B) GNPs. All of the GNPs are characterized by an irregular shape; in both samples, they are visible as aggregated and overlapping layered sheets and less often as a single sheet of a few layers. In particular, the higher transparency areas indicate the presence of single GNPs of a few layers, resulting from the effective graphite exfoliation, while the darker regions represent agglomerates of many GNPs. The tendency of GNPs to tangle together randomly, forming agglomerates, even with large dimensions, is more evident in the BOTTOM60 sample than in TOP60. The average measure of the lateral size of the individual TOP60 and BOTTOM60 GNPs, consisting of few-layers graphene planes, was estimated as <L> = 70 nm and <L> = 120 nm, respectively.

The TEM analysis indicates that the average size of GNPs is overestimated by DLS by a factor of 2.5 and 1.6 for TOP60 and BOTTOM60 GNPs, respectively, thus confirming the presence of small GNPs aggregates in water. On the other hand, also the sizes obtained from TEM images should be carefully considered: in this case, individual GNPs belonging to the aggregates cannot be measured and do not concur with the statistical analysis. 

An additional TEM investigation has been carried out on a 3L/SC sample, which is the commercial GNP reference in this study. In Supplementary Materials Figure S1, the TEM images show some peculiarities of the 3L/SC particles. Individual 3L/SC flakes have sizes of the order of 10^2^ nm, and their edges are peculiar. Several particles show a polygonal shape, with straight edges, thus suggesting that their borders are more ordered than the edges of TOP60/BOTTOM60, which instead confer to the particles a rounded shape.

### 2.3. UV-Vis Extinction Spectra of GNPs

When measuring the UV-vis light transmitted through a dispersion of GNPs, both the absorption (Abs) and the scattering (Sca) phenomena affect the amount of transmitted light leading to the measurement of the extinction (Ext) rather than a true absorbance [31,32]. Therefore, almost all of the experiments actually measure the extinction coefficient of the dispersed graphene particles. Ext is the sum of Abs (by the thinnest GNPs) and Sca (which mainly originate from the thickest GNPs or from the aggregates), as Ext = Abs + Sca [31]. The optical extinction spectroscopy of the well-dispersed and exfoliated nanoparticles in solution usually carries a lot of information about their size and thickness. The technique is also valuable when attempting to establish spectroscopic metrics [31], namely, a simple protocol aimed at obtaining quantitative determinations.

**Figure 4:**
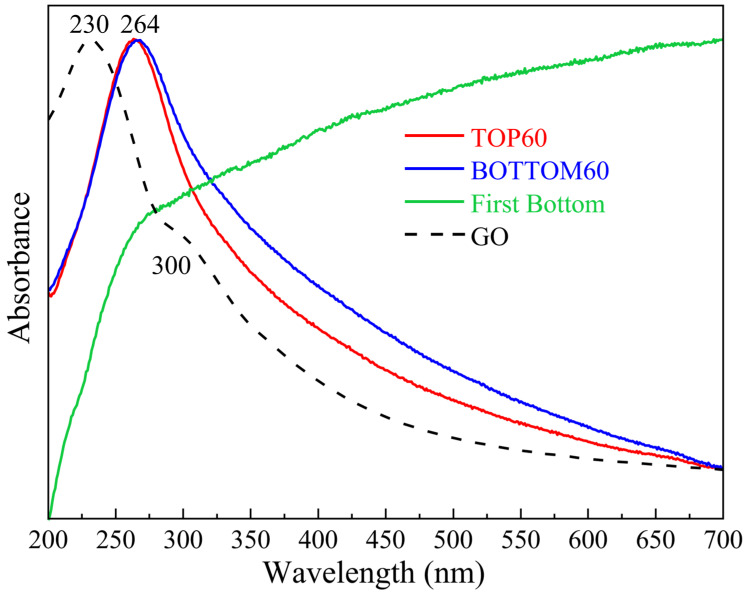
UV-vis normalized extinction/absorption spectra of the GNPs samples of TOP60, BOTTOM60, First BOTTOM and Graphene Oxide (GO).

Because the material we analyzed is made of rather thin GNPs, the observed UV-vis spectra present a broad and strong absorption feature peaking at about 270 nm, which is assigned to a strong intra-band transition characteristic of graphene and related materials [42,43,44,45,46]. Moreover, some amount of scattered light determines the overall background trend: as the particles’ average thickness gradually increases, the peak becomes wider. For very big particles, as for the First BOTTOM sample, the contribution of the scattered light dominates the spectrum (Figure 4). In Figure 4, the UV-vis extinction spectrum of the GNPs at different steps of production and Graphene Oxide (GO) are reported. Both for BOTTOM60 and TOP60 GNPs, we observe a strong absorption band at 264 nm, which is characteristic of well-exfoliated GNPs. This peak is wider for the BOTTOM60 than for the TOP60: the more pronounced asymmetric tail at longer wavelengths (blue curve) suggests that the BOTTOM60 particles are thicker and possibly functionalized (e.g., by oxidation); indeed, graphene oxide shows a broad asymmetric absorption in the same spectral range (purple dotted curve). Interestingly, the spectrum obtained from a sample of the First BOTTOM, namely the green curve shown in Figure 4, exhibits a distinct pattern. After the first centrifugation step, we separated the thicker nanoparticles, where the effective π-π stacking, due to attractive, non-covalent interactions between aromatic rings, is still present. Thick GNPs exhibit high reflectivity and diffusivity; thus, only a vanishing number of photons transmitted through the nanoparticles reaches the detector, and the scattering phenomenon dominates.

**Figure 5:**
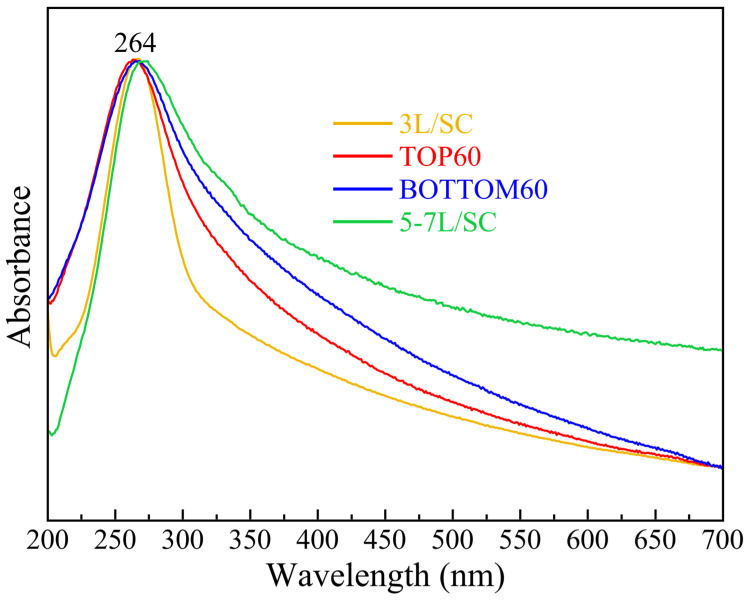
Comparison among normalized UV-vis absorption/extinction spectra of water dispersions containing commercial graphene nano-sheets (3L/SC, 5–7L/SC) and TOP60, BOTTOM60 GNPs.

The number of layers of the GNPs is estimated by comparing the experimental UV-vis spectra with those of commercial reference GNPs, which have a known average number of layers and/or using data reported in the literature for similar systems [31,32,45,46]. Conspicuous changes in the shape of the absorption peak as a function of <N> can be observed. In general, the ratio between the intensity of the long wavelength plateau (~550–800 nm) and that of the peak at 264 nm increases with the increasing thickness of GNPs [31,32]. In Figure 5, we compare the UV-vis spectra of TOP60 and BOTTOM60 with those of two commercial samples of few-layers graphene, 3L and 5–7L. To this aim, we prepared 3L/SC and 5–7L/SC water dispersions using the same ultrasound and centrifugation parameters adopted for the preparation of our GNPs samples in order to select particles with lateral sizes comparable to those of TOP60 and BOTTOM60. The spectra of TOP60 and BOTTOM60 lie between the curve of 3L and 5–7L samples, thus suggesting that the <N> value of TOP60/BOTTOM60 is less than seven layers. Of note, in the case of the commercial 3L sample (yellow curve in Figure 5), the width of the UV-vis peak spectrum suggests a narrower distribution of thickness. Moreover, as the number of layers increases (5–7L case), the position of the peak shifts slightly to higher wavelengths.

C. Backes et al. [31] developed a method for the determination of the <L> and <N> of liquid-exfoliated few-layer graphene nanosheets based on the ratio of the plateau value of the extinction coefficient taken at 550 nm and the extinction coefficient at the absorption maximum. They find the following linear relationship useful for the determination of <N>:

(1) <N> = 13.7 × ε550εmax− 1.2

Interestingly, this “metric” is consistent with our previous observations coming from the direct comparison with UV-vis spectra of commercial graphene nano-sheets, as shown in Table 2. According to Equation (1), we calculated the average number of layers of the TOP60 and the BOTTOM60 as four and six, respectively.

The stability of GNPs water dispersions was monitored during a time interval of about 6 months, during which TOP60 nanoparticles did not show signals of aggregation, while BOTTOM60 samples showed some aggregation but not of GNPs re-stacking since the average number of layers does not change with time. These conclusions are reached by means of UV-vis absorption experiments, which were conducted after 1 day, 30 days, 45 days, and 165 days (see Supplementary Materials Figure S2, Tables S2 and S3). Worth noting, we found a good reproducibility of the spectra over time, with negligible changes in the estimate of <N>, thus proving that re-stacking of TOP60 and BOTTOM60 GNPs does not occur. This is a key advantage of this preparation method, which combines a simple procedure and equipment, an environmentally friendly aqueous medium and control over the size and stability of the GNPs dispersion. 

### 2.4. Infrared Absorption Spectra of GNPs

**Figure 6:**
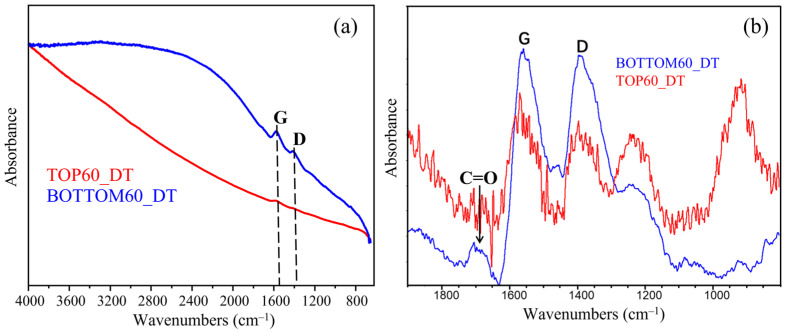
Normalized FTIR spectra of TOP60 and BOTTOM60 obtained in double transmission: (**a**) Spectra, as recorded; and (**b**) Spectra after baseline correction.

The FTIR spectra were recorded with a DT set-up on very thin films obtained by casting from a few drops of a GNPs water dispersion on an aluminum foil and dried in a vacuum chamber (Figure 6). Both spectra of TOP60 and BOTTOM60 show the IR active G peak close to 1600 cm^−1^ corresponding to the E_1u_ phonon of graphite observed at 1588 cm^−1^ and a strong and broad absorption feature in the range of the D Raman band. As shown by the spectra displayed after the baseline correction in the region 1800–900 cm^−1^, BOTTOM60 shows bands overall stronger and a different intensity pattern than TOP60. The stronger IR features of BOTTOM60, as observed in Figure 6a, could be rationalized considering that these GNPs are functionalized by polar groups [47,48], e.g., C=O, belonging to carboxylic groups. The chemical modification can be recognized thanks to the increase in the characteristic C=O stretching band at about 1700 cm^−1^, also often observed in other few-layer graphite samples [13,49,50] and functionalized graphene flakes obtained by means of ball-milling processes [36,37,47]. Polar functional groups grafted along the GNPs edges polarize the π electrons system of the graphene layers, and consequently, CC stretching vibrations acquire activity in IR.

**Figure 7:**
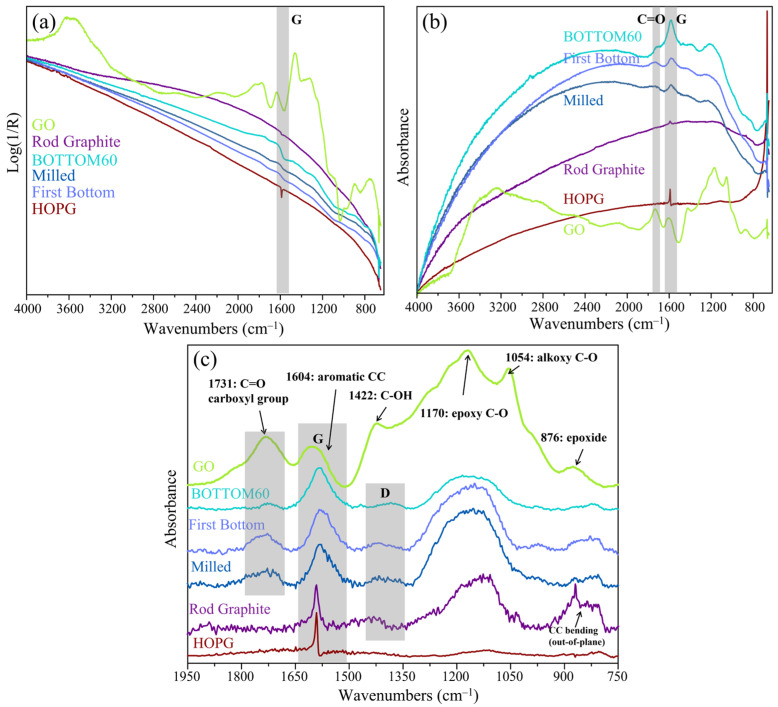
FTIR spectra of HOPG, ROD, GO, and GNPs samples at different preparation steps: (**a**) Specular reflection (SR) spectra; (**b**) absorption spectra from SR after Kramers–Kronig transformation; and (**c**) absorption spectra after baseline correction in the region 1950–750 cm^−1^.

The optical properties of carbon layers are strongly influenced by their morphology [18,28,51]. When the sample thickness does not allow a pure transmission measurement, the use of specular reflectance (SR) or diffuse reflectance (DRIFT) spectroscopic techniques provides information on the vibrational properties. We recorded the SR spectra, which can be transformed using the Kramers–Kronig (KK) relationship [33,34], thus obtaining good-quality absorbance spectra. To the best of our knowledge, the IR–SR set-up adopted here has never been reported in the literature to record the IR spectra of GNPs samples. The thickness and the flatness of the sample are crucial for SR measures; if the sample is too thin, some light transmitted through the sample and reflected by the substrate (as for the DT technique) could reach the detector together with the light reflected by the surface. A spectrum simultaneously containing information coming from light absorption and reflection is hardly interpretable [35]. We performed SR measurements on a relatively thick film prepared by the drop casting of GO, and BOTTOM60 aqueous dispersions, whereas the high dilution of the TOP60 fractions did not allow for the preparation of films thick enough to record an SR spectrum. For Highly Oriented Pyrolytic Graphite (HOPG) and ROD, the SR spectra were recorded directly on the surfaces. In Figure 7, we report the SR FTIR spectra—before and after KK transformation—for HOPG, ROD, GO, and GNPs at different steps of the preparation process. In Figure 7b, the KK transformed spectra are shown after a baseline correction in order to make the comparison effective. All of the spectra show a G-like IR band, which displays a clear evolution in width and relative intensity from HOPG to GO. IR analysis by means of SR measurements for GNPs prepared at different times of the ball milling process was also carried out and illustrated in Figure S3. We observe that GNPs undergo functionalization under the grinding and exfoliation action of the ball milling in air. In Supplementary Materials Table S4, the increasing intensity ratios of the C=O stretching band and G-band for increasing ball milling times are reported.

As shown in Figure 7c, a sharp G band is exhibited for an ideally infinitely extended graphene sheet (HOPG and ROD spectra). As mechanical milling and high-energy ultrasound are applied (first bottom), the G peak broadens, and the bands assigned to the carbonyl/carboxyl groups increase in the IR spectra. After the second centrifugation, a smaller fraction of functional groups remained in the BOTTOM60 sample. This is shown by the data reported in Table 3: the area ratio of C=O to G band of the First BOTTOM it is AC=O/AG = 0.43, while for the BOTTOM60, it is AC=O/AG = 0.11. It is worth noting that the degree of functionalization of BOTTOM60 nanoparticles is completely different from that of GO; indeed, TOP60 and BOTTOM60 contain fewer and almost only C=O functional groups, showing that selective oxidation can take place through the physical methods we adopted. The chemical acid–base titration [52,53,54] carried out on the samples of GNPs confirmed the presence of some -COOH functional groups in the TOP60 and BOTTOM60 GNPs.

In addition to the C=O stretching and G bands, all of the samples, with the exception of HOPG, show IR absorptions in a frequency range close to the Raman D band (near 1350 cm^−1^), which, by analogy, could be tentatively ascribed to the collective ring breathing modes. Moreover, a strong and broad IR absorption, covering the 1250–1050 cm^−1^ region, is clearly displayed in all of the IR spectra of Figure 7c, with the exception of the spectrum of HOPG, where it appears as a very weak and broad feature. To the best of our knowledge, the observation of this IR absorption feature in graphitic materials has not been discussed so far in the literature. Interestingly, the strong band in the 1250–1050 cm^−1^ range is already present in the spectrum of ROD, together with the sharp feature at 870 cm^−1^, corresponding to the transition of the out-of-plane A_2u_ **q** = **0** IR active phonon of graphite19, while both these absorptions are very weak in the HOPG spectrum. The lack of the A_2u_ transition in the spectrum of HOPG is a consequence of the orthogonality between the electric field vector of the IR beam (which is parallel to the graphene sheets in our experimental set-up, with light beam incidence orthogonal to the basal plane) and the transition dipole moment of the 870 cm^−1^ vibration, which is orthogonal to the graphene plane. By analogy, we infer that also the broad feature in the 1250–1050 cm^−1^ range is sensitive to the orientation of the crystalline domains, which is instead isotropic in synthetic graphite, namely in ROD or in the thick films of GNPs. It is worth noticing that a similar, very strong, and broad absorption band appears in the same region of the spectrum of graphene oxide, showing a more structured shape with peaks and shoulders floating on it, which are commonly assigned to vibrational modes localized on various chemical groups containing oxygen (see Figure 7c). The broad and strong background absorption observed for GO could be due to the same phenomenon responsible for the corresponding IR band of synthetic graphite and of GNPs. Further investigations may be deserved for a careful vibrational assignment for this strong IR absorption, but it is beyond the scope of this work.

### 2.5. Raman Spectra of Graphene-Based Nanoparticles (GNPs)

Raman spectroscopy is by far the most used technique for the structural characterization of graphite and graphene, as well as nano-structured carbon-based materials, e.g., carbon nanotubes and graphene nanoparticles [18,19,20,21,22,27,28,29,30,31,32,55,56,57,58,59,60,61,62,63,64,65,66]. 

The following Raman analysis of our GNPs, comprehensive of a thorough comparison with the Raman spectra of some reference materials, has a twofold aim. The first goal is to assess whether the Raman patterns of TOP60 and BOTTOM60 are consistent with the information extracted by means of the previous analyses (Section 2.1, Section 2.2 and Section 2.3). As a second step, we provide additional insight into the structural differences between the two GNPs samples and their distinctive characteristics with respect to commercial GNPs samples (3L).

The discussion of the Raman data is organized as follows:

Section 2.5.1 reports a general description of the Raman features (first- and second-order Raman spectrum) of carbon-based materials characterized by the presence of graphene-like domains, ranging from highly ordered graphite to graphene and GNPs. This Section is devoted to providing the reader with the theoretical framework and with the available information that forms the ground for the interpretation of the Raman spectra of the TOP60 and BOTTOM60 samples.

Section 2.5.2 presents the Raman analysis we carried out on some commercial materials: the pristine synthetic graphite used for preparing GNPs and a commercial sample made of thin graphene-nanoparticles with <N> = 3. This analysis provides reference Raman spectra, which allows shedding light on the structural peculiarities of TOP60 and BOTTOM60, as displayed by their Raman response.

Section 2.5.3 and Section 2.5.4 deal with the Raman spectra, recorded with λ_exc_ = 633 nm, of TOP60 and BOTTOM60 and discuss analogies and differences from the commercial 3L sample. In Section 2.5.5, we report a deepening of the Raman analysis by means of the spectra recorded with excitations of λ_exc_ = 532 and 405 nm. Section 2.5.5 ends with a list of observations that have been collected through the detailed analysis of the parameters from the curve fitting of the Raman spectra (the deconvolution/curve fitting procedure is reported in detail in SI). Here, below, we will briefly illustrate the general procedure we adopted while carrying out the spectra deconvolution.

The curve fitting has been performed in a systematic way, searching for a solution (deconvolution) with a minimum number of individual components (bands). The components were selected according to the direct analysis of the Raman spectral pattern, often showing structured bands and the vibrational assignment from the literature. The fitting allows obtaining the peak position and peak height, the integrated areas, and the Full Width at Half Maximum (FWHM) values of the individual band components. Due to the intrinsic limitation of the experimental set-up and the complex nature of the samples, we could not determine reliable absolute Raman Cross-Sections; therefore, the band intensities normalized to the G band will be analyzed according to a consolidated practice [31].

**Figure 8:**
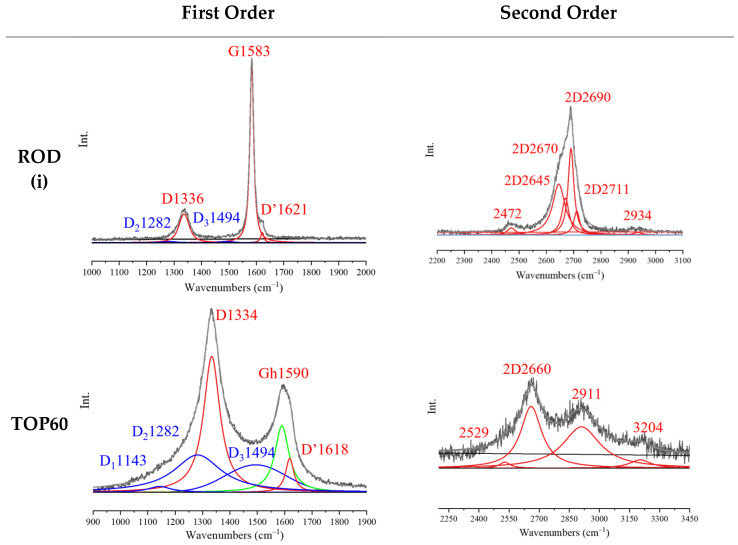
Result of the curve fitting (deconvolution) of the Raman spectra (λ_exc_ = 633 nm) of ROD(i) sample and of TOP60 sample.

Figure 8 shows the graphical result of the curve fitting for two representative examples, namely the Raman spectra (λ_exc_ = 633 nm) of the ROD sample, which maintains the features of crystalline graphite, and of the TOP60 sample, which shows remarkable changes in the spectrum, because of the reduced sizes of the GNPs and of their peculiar morphology.

Interestingly, probing ROD at different points shows that the sample is not structurally homogeneous on the micrometer scale, and it consists of an assembly of graphite domains characterized by different amounts of disorder. Here, we selected the ROD (i) spectrum as representative of the ROD sample; however, the results obtained by two different measurements, ROD (i) and ROD (ii), are illustrated in the Supplementary Materials for the sake of comparison.

In Table 4, we report a summary of the relevant parameters obtained through the deconvolution of the whole set of the spectra recorded at 633 nm, while in Supplementary Materials (Figures S4–S12 and Tables S5–S13), we collected the Raman spectra at three excitation wavelengths (λ_exc_ = 633, 532, 405 nm) for all the samples listed in Table 4, together with the result of the fitting.

#### 2.5.1. Raman Spectra of Graphite/Graphene and GNPs: General Features and Theoretical Background

In this paragraph, we summarize some general characteristics of the Raman spectra of graphite and related materials, the commonly accepted interpretation of some remarkable features, as well as the theoretical concepts which enabled the vibrational assignment and the interpretation. We mainly focus on the topics that are relevant to the discussion of the spectra of our GNPs.

All of the materials containing extended graphene-like domains show a G line close to 1582 cm^−1^, corresponding to the excitation of the doubly degenerate E_2g_ phonon at the Brillouin zone center (phonon wave-vector **q** = **0**), which is associated with the CC stretching vibrations of the graphite lattice [19] (Supplementary Materials Sketch S1). The low-frequency, very weak E_2g_ band (shear mode, 42 cm^−1^) and the G line correspond to the only two Raman transitions allowed in the first-order Raman spectrum for perfect graphite crystals [19]. The FWHM of the G line is affected by the degree of disorder of the sample, and its broadening is often taken as evidence that the sample consists of a distribution of graphene-like domains of different sizes/shapes [31,58].

The appearance of the D band close to 1350 cm^−1^ is ascribed to the activation of forbidden transitions of the ideal perfect graphite/graphene crystal, corresponding to phonons with a wave-vector near the Brillouin zone edge at the **K** point, and correlated to the highly symmetric **q** = **K** phonon (ring breathing mode, see Supplementary Materials Sketch S1). The activation mechanism, first described by Thomsen et al. [63] and referred to as “double resonance”, exploits the symmetry breaking associated with some structural/chemical disorder. It is very selective because it is a resonance phenomenon. Indeed, the resonance condition, i.e., the matching between the photon energy of the exciting laser and the energy of a given vertical π-π* electronic transition, allows for the enhancement of the Raman cross-section of a selected phonon, with a proper **q** value in a region near to **K** point, thus explaining the observed frequency dispersion of the D line with the exciting laser wavelength.

The edges of the graphite sheets represent a peculiar kind of structural defect, which determines the confinement of the π electrons in domains with a finite size. Other possible structural defects originate by disrupting the packing order of graphene layers (turbostratic graphite), by the exfoliation, by the formation of holes, sp^3^ carbon atoms, or by chemical functionalization, occurring along the edges and/or located in the inner regions of the graphene networks. For the above reasons, the intensity ratio (ratio of the peak heights) ID/IG is commonly used for estimating the overall degree of the disorder [27,28,55,59], irrespective of the kind of defects induced by the treatments on the graphite specimens.

In the presence of edges, a feature called the D’ line appears on the higher wavenumbers side of the G line. The intensity ratio of ID’/IG is a specific marker of graphene edges since D’ is assigned to the CC stretching vibrations localized on the CC bonds located on the periphery of the graphene planes. Some authors suggested that a correlation exists between ID/IG and ID’/IG, which allows for the classification of the kind of disorder of the basal planes mainly due to edges or structural defects of another kind, such as sp^3^ defects, vacancies, or chemical defects [64,65]. Small ID/ID’ values correspond to samples with a large number of peripheral CC bonds, which is typical of small GNPs, mainly consisting of integer graphene-like layers.

While approaching nano-sized graphene-like domains, both the peculiar electronic structure of each domain/particle and the size/shape distribution of domains/particles in the sample modulate the peak position and intensity of the D band through a variety of different resonance conditions of the Raman modes. Such a complex situation requires a multi-wavelength Raman approach for a thorough study, and it will be adopted in our analysis. Moreover, graphene/graphite particles of finite size can present normal modes which do not match with any phonon of the perfect crystal and are possibly not affected or scarcely affected by resonance. These localized vibrations can give rise to additional Raman transitions.

The second-order Raman spectrum of graphite and graphene shows a strong transition near 2700 cm^−1^, which is usually referred to as a 2D band since it corresponds to the two phonons transition associated with the **q**~**K** phonons, which are responsible for the D line. The 2D transition also allows for an ideally infinite graphene sheet or graphite crystal, and it exploits the resonance enhancement phenomena [18,19]. Parallel to the increased D band, size-induced confinement and exfoliation are responsible of some modulation of the 2D Raman feature [27,66]. While graphite shows the very characteristic asymmetric shape of the 2D band, with a sharp peak at 2688 cm^−1^ (at λ_exc_ = 633 nm) and a broader feature at smaller wavenumbers, structural defects as the confinement, exfoliation, or disorder in the layers stacking determine a more symmetric shape of the peak and, sometimes, provoke the intensification of other distinct phonon overtones and combination bands at higher frequencies. The ratio between the 2D and G band intensities (I2D/IG) often decreases in the presence of disorder. This decreasing trend with the structural disorder is typical of a regime characterized by small graphitic domains, while in the case of large graphene sheets (with a vanishing small D band), the intensity of I2D/IG is a marker of the number of stacked sheets, and it increases as the number of layers in the stack decreases. Indeed, for a single layer (graphene), I2D/IG = 4 at λ_exc_ = 532 nm, while it approaches the typical value of graphite (I2D/IG = 0.5) as far as the number of staked layers increases [27]. Interestingly, the characteristic graphite-like shape of the 2D band, showing a sharp component on the higher frequency side, is quickly recovered [27], already for N > 5. 

For the above reasons, in the case of GNPs, the interpretation of the trend of I2D/IG should be undertaken considering the competitive effect of the exfoliation (which, in principle, should increase 2D intensity) and of the breaking of the basal plane (which, instead, decreases the 2D band). As for the shape of the 2D band, it results from the balance between the contributions by the regular AB stacking of graphene layers, which concur to the increased sharp graphite-like component, and by turbostratic stacks and/or a few layers of flakes regularly stacked (N < 5), which contribute to the lower-frequency component with a symmetric shape.

In addition to the very strong 2D features, the second-order Raman spectrum of graphite shows two other very weak bands (~2450 and ~3250 cm^−1^) associated with two quanta vibrational transitions. Different interpretations of these findings are reported in the literature, based either on the appearance of the strong features of the vibrational density of states [19] or on specific vibrational assignments to overtones/combination of **q** ≠ **0** fundamental modes [18].

#### 2.5.2. Raman Spectra of Commercial 3L GNPs

**Figure 9:**
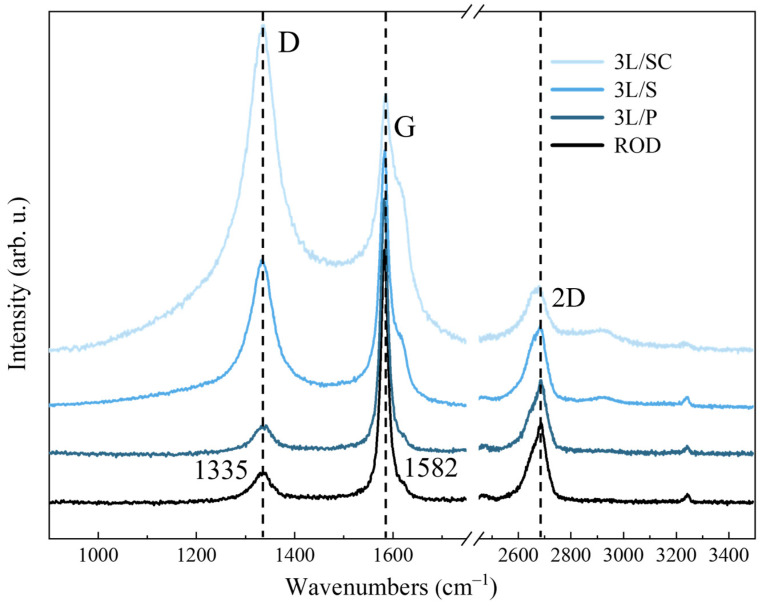
Raman (λ_exc_ = 633 nm) spectra of the pristine synthetic graphite sample (ROD) and of the commercial 3L GNPs (3L/P: the pristine sample; 3L/S: the sample after sonication in water; 3L/SC: the sample after sonication and centrifugation).

To study how the ball milling and the sonication processes change the graphite structure during the preparation of our GNPs samples, we first characterized the commercial 3L sample, which will be considered a reference material to be used for the sake of comparison. We recorded the Raman spectra of the three different samples, namely: (i) the sample as received (pristine 3L, hereafter referred to as 3L/P), consisting of very large (tens of micrometers) flakes; (ii) the sample recovered after sonication in water (3L/S) consisting of a powder of smaller flakes, and (iii) the nanoparticles (3L/SC) obtained after the same sonication procedure as 3L/S followed by centrifugation, according to the procedure adopted for the herein discussed TOP 60. The Raman spectra of the 3L samples, obtained at λ_exc_ = 633 nm, are reported in Figure 9, together with the spectrum of the ROD, which was used as the raw material for the preparation of the GNPs. Interestingly, 3L/P shows a Raman spectrum similar to that of the ROD sample. At first sight, this similarity is quite surprising, but it can be justified by the observations below.

Synthetic graphite consists of a mixture of graphite microcrystals showing AB stacking order and graphite domains characterized by a turbostratic arrangement of the graphene layers.

Thin, exfoliated graphene flakes (3L/P) are probably a mixture of ordered large sheets, which form few-layer stacks, and smaller few-layer particles, possibly showing some amount of turbostratic disorder.

The 2D line shape is, in principle, sensitive to the thickness of the graphite flakes, and a symmetric band shape is expected for very thin flakes. Therefore, the presence of some large and thick flakes in 3L/P is responsible for the observed sharp graphite-like 2D feature. In contrast, very thin flakes of 3L/P, as well as turbostratic flakes, give rise to the symmetric component. This component has a shape similar to that one resulting from the turbostratic domains in ROD graphite.

The spectrum of 3L/P shows a relatively small D line, accompanied by a weak D’ shoulder, which proves that the lateral size of most of the flakes is large; thus, the intensities of the Raman transitions related to the edges are relatively low.

The spectrum of 3L/S shows that sonication has a remarkable effect: the D and D’ intensities increase, and the G peak becomes broader (both ID/IG and ID’/G are about three times larger, passing from 3L/P to 3L/S). In addition, a new G component was observed, namely the weak G_h_ at 1590 cm^−1^, and some new broad components in the region of the G and D bands. According to spectral deconvolution (see Supplementary Materials Figures S5–S12, Table 4 and Tables S5–S13), these new features can be described as three broad bands, hereafter referred to as D1, D2, and D3. In addition, the 2D band almost loses its sharp component; moreover, a component corresponding to a combination band (G + D) at about 2900 cm^−1^ appears (see Supplementary Materials, Figure S10 and Table S10).

The Raman pattern of 3L/SC changes more significantly: (i) the D’ and D lines further increase; (ii) the G_h_ peak is the dominant feature in the G region; (iii) the D1, D2, and D3 bands grow; (iv) the 2D band becomes more symmetric and down-shifts with respect to both 3L/P and 3L/S (see SI, Tables S7–S9), and (v) the intensity of the G + D band increases. We reasonably ascribe the variations observed to the confinement induced by the remarkable reduction in the average lateral dimension of the particles sorted by centrifugation.

#### 2.5.3. Raman Spectra of TOP60 and BOTTOM60

**Figure 10:**
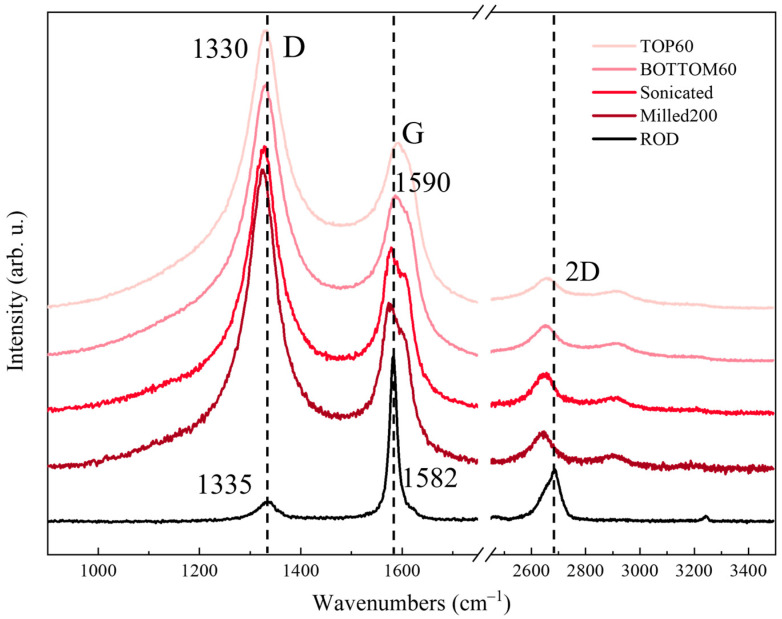
Raman spectra (λ_exc_ = 633 nm) of GNP samples at different preparation steps.

The previously observed behavior of the Raman spectra of the 3L samples parallels the evolution of the Raman features of our GNPs, as reported in Figure 10, where the TOP60, BOTTOM60, Milled200, and ROD spectra are compared.

In Figure 10, the spectrum of the ROD sample is dominated by the G band. The D band has a very low intensity because of the presence of large graphite domains and low disorder.

The appearance of a strong D band in the Milled200 spectrum, with a peak intensity clearly exceeding the G line, indicates a remarkable increase in the disorder (edges formation) due to the breaking of the graphite crystals into smaller fragments.

However, milling can induce a variety of other structural defects through the exfoliation and breaking of the graphene sheets. Moreover, the breaking of the covalent CC bonds can promote structural rearrangement with the formation of localized defects, e.g., holes and sp^3^ carbon atoms. If the treatment is performed in air, water, or in the presence of additives, the occurrence of chemical modifications (e.g., oxidation, as observed by IR analysis of the Milled200 sample) is expected [33,34].

After the second centrifugation, we selected small GNPs, which have average lateral dimensions of <L> = 70 nm (TOP60) and <L> = 120 nm (BOTTOM60), a slightly different thickness, according to TEM observations, and different edges functionalization according to IR measurements.

Let us compare and comment in more detail on the spectra of the TOP60 and BOTTOM60 GNPs:

(a) The TOP60 and BOTTOM60 nanoparticles show quasi-superimposable Raman spectra, with strong D and D’ lines, as expected for small size (small <L>) particles. The G line is now a broad and symmetric peak at higher wavenumbers with respect to HOPG and ROD and coincident with the G_h_ component of 3L/SC. The similarity of the GNPs spectra confirms that the Raman analysis is sensitive to the drastic reduction in <L> to the scale of the tenths of nanometers, but it seems poorly sensitive to the differences in the average lateral size, which has been highlighted by other techniques. In addition, it does not provide specific information about GNPs thickness nor on the chemical functionalization of the edges, which is indeed more abundant for the BOTTOM60 samples, according to IR evidence. As will be illustrated in Section 2.5.5, a more detailed description can be reached only by means of quantitative analysis based on the curve fitting of multi-wavelength Raman spectra.
(b) The D1, D2, and D3 bands rise, showing a higher intensity with respect to 3L/SC, thus suggesting the presence of additional disorder, probably associated with the local structure of the edges.
(c) The highly symmetric shape of the 2D band demonstrates that the stacking in BOTTOM60 and TOP60 could be affected by turbostratic disorder.
(d) We observe several transitions involving two vibrational quanta in addition to the 2D line, in particular of the strong G + D combination band, which is a typical molecular feature observed in the Raman spectra of polycyclic aromatic hydrocarbons, PAHs [67,68]. It can be taken as evidence that we are approaching the molecular regime. As already observed, this feature was clearly observable also for 3L/S and 3L/SC.
(e) As in the case of PAH molecules [67,68], the intensity ratio I2D/IG is remarkably lower than in graphite. Tentatively, we ascribe such behavior to the small lateral size of our particles.

In this respect, it is instructive to compare our result with those reported in [31], where an increasing trend of I2D/IG has been observed for samples made of graphene particles while passing from thicker to thinner GNPs: on this ground, the authors [31] provide an empirical relationship between <N> and I2D/IG, which can be exploited to obtain the value of <N> from the Raman measurements (at 532 nm). Even if our TOP60/BOTTOM60 GNPs are, on average, very thin (according to evidence from the UV-vis absorption spectra), their I2D/IG ratio is rather low, yielding a predicted value of <N> ~ 56 and <N> ~ 118 for TOP60 and BOTTOM60, respectively, (according to Equation (6), proposed by [31]). This result indicates that the ratio I2D/IG is affected in an entangled way by the number of sheets in the stacking and by other structural characteristics resulting from the method of preparation of GNPs; for these reasons, the use of I2D/IG parameter for defining a general metric requires great caution.

#### 2.5.4. Raman Spectra of TOP60 vs. 3L/SC

**Figure 11:**
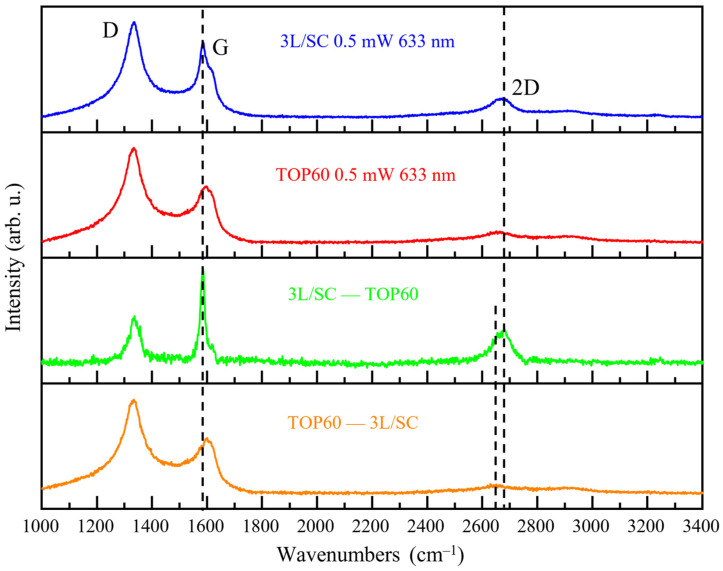
From the top to the bottom: Raman spectra of 3L/SC, TOP60, and subtraction spectra (3L/SC—TOP60) and (TOP60—3L/SC). Raman spectra were recorded with λ_exc_ = 633 nm, with 0.5 mW laser power.

In Figure 11, we directly compare the Raman spectra of 3L/SC and TOP60, both showing lateral dimensions in the range of 10–10^2^ nm and similar thickness; from these two spectra, we can build a subtraction spectrum (3L/SC–TOP60) and the complementary subtraction (TOP60–3L/SC), which highlight some interesting differences between the two samples. TOP60 has, on average, a more disordered structure with respect to 3L/SC: indeed, the subtraction (3L/SC–TOP60) shows a “more graphite-like” Raman pattern, with a shaper G line and a rather strong 2D peak, together with a “flat” background in the D g region (almost no contribution by D1, D2, and D3 bands).

Generally speaking, the increased disorder in TOP60 could be due to the presence of more disordered and/or irregular edges, different from those of graphite (step edges), which mainly present zigzag or armchair profiles [25,69,70]. This feature justifies the presence of larger D1, D2, and D3 components in TOP60, and it can also be responsible for the turbostratic-like stacking of the sheets. The occurrence of more regular edges in 3L/SC, which has also been inferred by the TEM images (Supplementary Materials, Figure S1) is compatible with the softer preparation procedure (no mechanical milling). The higher intensity of the 2D band of 3L/SC could be ascribed to a more regular stacking of the sheets in this sample. Once again, we achieved further evidence of the difficulty of extracting information about <N> straightforwardly from the 2D features.

It is worth noticing that the conclusion reached by means of the spectra subtraction is perfectly coherent with the picture from curve fitting analysis (Supplementary Materials, Figures S10 and S11 and Tables S10 and S11), showing that the G line profile of 3L/SC requires that two different components, namely G and G_h_ are included in the fitting, at 1582 cm^−1^ (G line of graphite) and at 1590 cm^−1^ (G component of the TOP60 = G_h_), respectively.

#### 2.5.5. Multi-Wavelength Raman Spectra

**Figure 12:**
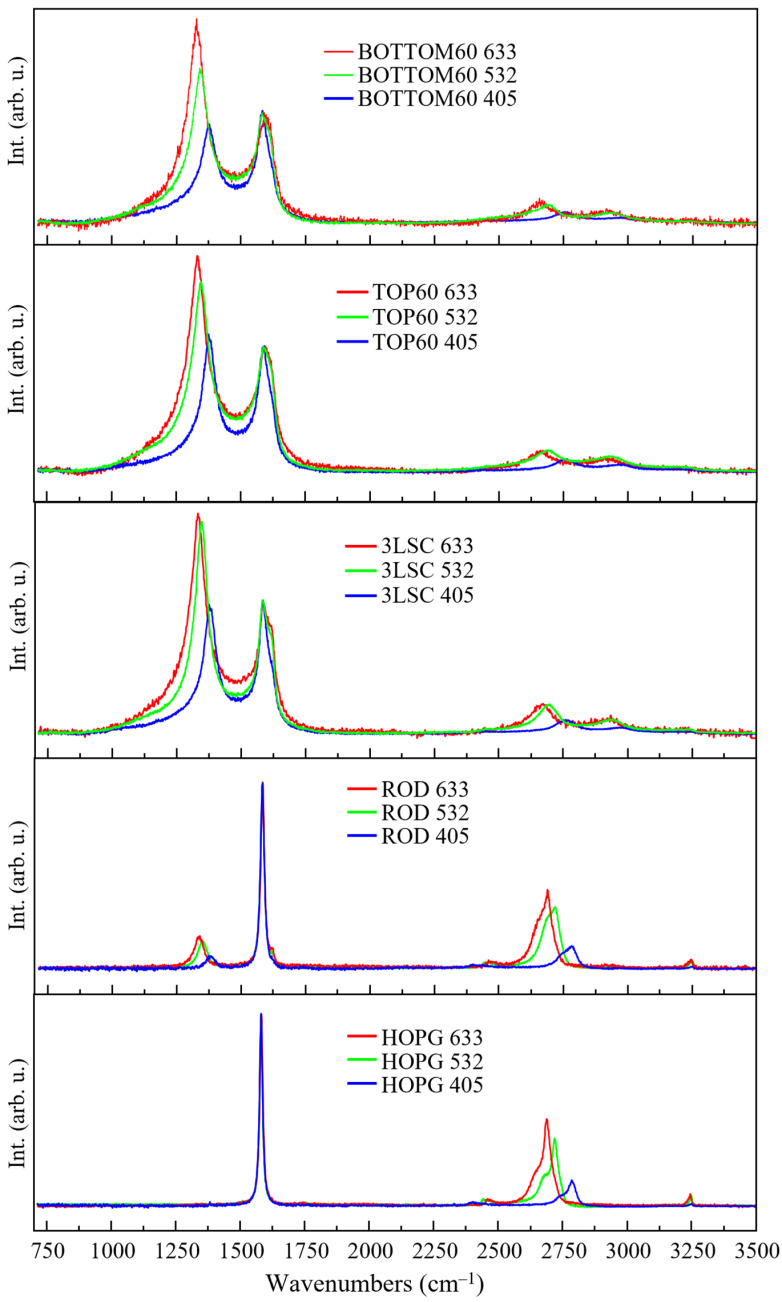
Multiwavelength Raman spectra (λ_exc_ = 633, 532, 405 nm) of: TOP60, BOTTOM60, 3L/SC, ROD, HOPG samples.

In Figure 12, the Raman spectra of HOPG, ROD, 3L/SC, TOP60, and BOTTOM60 recorded at 633 nm, 532 nm, and 405 nm acquired in the same sample position are reported.

The Raman features observed in the spectra recorded at 532 nm and 405 nm are consistent with the conclusions reached by analyzing the spectra obtained with a 633 nm excitation. However, as previously discussed, the effects of resonance give rise to a rather complex scenario, which widens the information on the size/structure of the GNPs samples vs. the reference materials.

The spectra obtained with an excitation at 633 nm better highlight the differences between TOP60 or BOTTOM60 and 3L/SC, which we ascribed to some different kind and number of structural defects, possibly involving the edges, as suggested in the previous section. Interestingly, when we excite with a laser at a shorter wavelength, the first-order Raman spectra of the three GNPs samples look more similar (Supplementary Materials Figure S4), while some differences between TOP60 and BOTTOM60 are revealed in the 2D region in the spectra obtained with an excitation at 532 nm.

As firstly observed by Pocsik [57] by means of a multi-wavelength Raman investigation of microcrystalline graphite samples, the D line shifts towards higher wavenumbers, while the resonance is tuned with higher energy electronic states. The 2D bands follow the same trend, showing an upward shift about two times larger with respect to its corresponding fundamental transition responsible for the D line. Our GNP samples adhere to this trend (Figure 12). Moreover, the intensity of the D band changes with the change in the laser wavelength, following the same trend for the three GNP samples, showing a decreasing D line intensity while the excitation energy increases.

The detailed analysis enabled by the deconvolution of the Raman spectra of TOP60, BOTTOM60, 3L/SC, 3L/S, 3L/P, ROD, and HOPG is illustrated in SI, where the derived parameters (frequencies and intensities of the band components) are reported (see Tables S5–S13) for the spectra recorded at the three different exciting wavelengths. Here, we summarize some remarkable trends highlighted by this analysis, mainly focusing on TOP60 and BOTTOM60 (some relevant parameters from the spectra fitting are reported in Table 5).

From the DLS, TEM, and UV-vis spectra, we inferred that TOP60 and BOTTOM60 have comparable lateral sizes and a number of layers, but, on average, BOTTOM60 particles are slightly larger and thicker. In the following, we discuss whether this finding is consistent with the Raman results.

(i) Overall, the Raman pattern and the parameters extracted by the fitting confirm that the degree of structural disorder, which is related to the particle size, is comparable for TOP60 and BOTTOM60. However, some differences can be appreciated by looking at the peaks’ intensity ratios ID/IG, which are reported in Table 5. The spectra recorded with laser wavelengths of 532 nm and 405 nm show that BOTTOM60 has lower ID/IG values than TOP60, and the same scenario results from an alternative measure of the disorder, namely from the ratio among areas of D and G components, AD/AG (Table 5). These data agree with the finding that TOP60 GNPs are smaller, meaning that the confinement effects associated with the presence of the edges are more effective. This evidence is further supported by the ID’/IG (and AD’/AG) values, which indicate a more important contribution from the vibrational modes localized on the GNPs edges in the case of TOP60. In this framework, it seems difficult to rationalize the apparent anomaly of the ID/IG values obtained at 633 nm excitation, which suggests the opposite trend, showing a smaller value (2.04) for TOP60, compared with ID/IG = 2.30 for BOTTOM60 (see Table 5). The analysis of ID/ID’, point (ii) below, offers an interpretation of it.
(ii) The ID/ID’ parameter, which is often used to assess the relative amount of edges and other kinds of structural disorders, shows a rather systematic trend at each fixed excitation wavelength while passing from 3L/SC, TOP60 and BOTTOM60. For L/SC ID/D’, the values are 3.8 (633 nm), 4.3 (532 nm), and 5.2 (405 nm). These values are close to those reported in Table 5 for TOP60. A higher ID/ID’ value is always found for BOTTOM60 (see Table 5), thus suggesting that in the spectra of BOTTOM60, the contribution to the disorder due to the presence of edges is smaller. This finding explains the apparent anomaly of ID/IG at 633 nm (point i above). The large ID/IG value of BOTTOM60 could be ascribed to the presence of a structural/chemical disorder not associated with confinement by edges, which seems to be efficiently probed while exciting in the red.
(iii) Different from the D component, the frequencies of the broad D1, D2, and D3 components turn out to be independent of the excitation energy. This behavior means that the D1, D2, and D3 components correspond to the Raman transitions not affected or scarcely affected by resonance: some of them can be ascribed to localized vibrations, which are not coupled with low-energy π electron transitions.
(iv) Interestingly, the intensity of D1, D2, and D3 is scarcely affected by the kind of nanoparticles, and it shows a decreasing trend with the decreasing of the exciting laser wavelength. This finding confirms that the D1, D2, and D3 components can be ascribed to the localized vibrations of the disordered regions, possibly in the presence of carbon atoms in sp3 hybridization and/or to vibrations involving functional groups (C=O) on the GNPs edges.
(v) GNPs show a D + G peak with an intensity comparable to that of the 2D line: this feature is indicative of the small size of the TOP60 and of BOTTOM60 GNPs since it is hardly observed in the case of large graphene sheets, while it is a remarkable feature in the second-order Raman spectra of large PAH molecules [67,68].
(vi) The A2D/AG (or I2G/IG) values (Table 5) highlight some differences between TOP60 and BOTTOM60. While they are insensitive to the GNP type while exciting with the blue laser, I2G/IG shows an opposite trend for the Raman experiments using 633 nm (a higher value for BOTTOM60) and 532 nm excitations, where TOP60 shows the highest value. These trends are consistent with the tendency of the A2D/AG values. As already mentioned, 2D band intensity is often referred to as a parameter sensitive to the graphene layers’ stacking, but the observed trends confirm that the use of this parameter presents a delicate issue since it is affected by several factors, which in turn depend on the resonance phenomena.

#### 2.5.6. GNP Structure Modifications upon the Photons Flux

**Figure 13:**
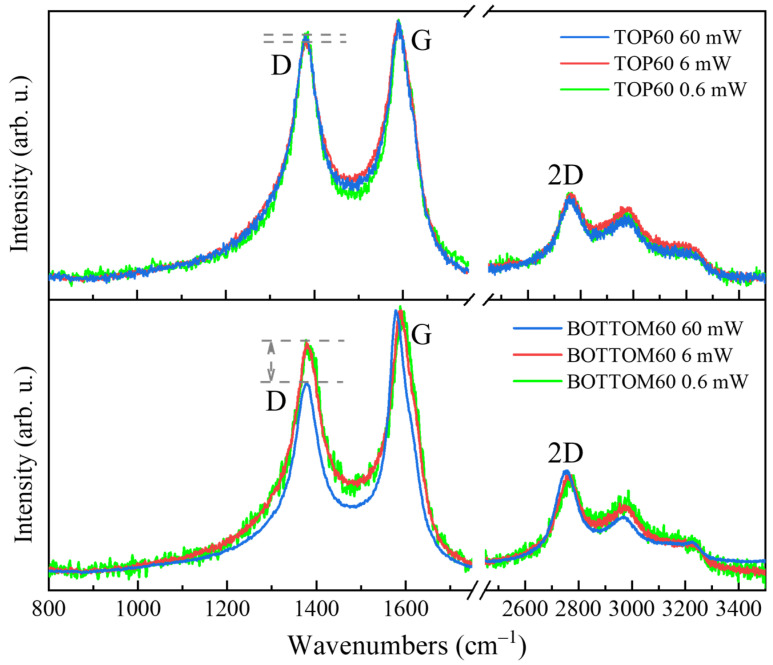
Raman spectra of the GNPs samples of TOP60 and BOTTOM60 excited by blue (λ_exc_ = 405 nm) laser at different power values at the same point.

An irreversible change in the Raman features for BOTTOM60 and TOP60 can be observed while illuminating the sample with an intense laser beam. The phenomenon depends on the power of the laser, as illustrated in Figure 13 for TOP60 and BOTTOM60, both excited with a 405 nm laser (other examples for different laser lines are illustrated in Supplementary Materials, Figure S13).

It was observed, especially in the case of BOTTOM60, a remarkable and irreversible change in the D band that weakens while increasing the power of the laser beam from 0.6 mW to 60 mW (Figure 13, bottom panel). We suggest an explanation by considering the UV-vis absorption spectrum of the GNPs, which shows a broad asymmetric tail of the main UV-vis peak at 260 nm towards a long wavelength. The effective absorption of the laser photons by GNPs could be responsible for the localized heating due to the inefficient thermal dissipation because of the small size of the particles. The temperature increase, in turn, favors structural rearrangement.

The observation that GNPs rearrange under photon flux can also explain the small differences observed in the spectra of the same sample recorded focusing the laser beam at different positions (Supplementary Materials Figure S14). Indeed, the structural rearrangement induced by the photons could be more or less effective from point to point because of the slightly different photon density deposited on the surface, which cannot be perfectly controlled. The remarkable conclusion is that during the Raman characterization of GNPs, the probe can modify the sample to some extent.

In order to collect reliable data, it is thus mandatory to minimize this effect. We adopted a strategy of performing some test experiments on different points of the sample in order to determine the maximum power of the laser, which does not induce detectable rearrangements. Each Raman spectrum must be further validated by means of a second measure (focusing on the same point, with the same set-up), which can reveal changes in the Raman response, possibly induced during the first Raman scan.

## 3. Materials and Methods

### 3.1. Preparation Procedure of Graphene-Based Nanoparticles (GNPs)

#### 3.1.1. Materials

As a starting material, we used a synthetic pristine graphite rod (496561-240.5G), hereafter referred to as ROD, containing a very low number of non-carbon impurities (99.995% carbon), purchased from Sigma-Aldrich, (Schnelldorf, Germany). Commercial 3-layer (900561-500MG) and 5–7-layer (799084-500MG) GNPs samples, hereafter referred to as 3L and 5–7L, respectively, were purchased from Sigma-Aldrich. Molecular biology reagent grade water (W4502-1L) from Sigma-Aldrich was used to prepare the GNP water dispersion.

#### 3.1.2. GNPs Preparation

**Scheme 1:**
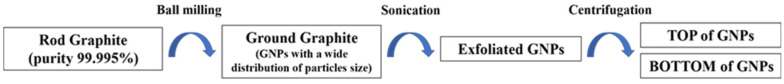
Diagram of the GNPs preparation process.

Two types of GNPs were prepared in this work following a procedure reported in the diagram of Scheme 1.

Ball milling procedure: The ROD was weighed in quantities of 500 mg and inserted into a stainless-steel jar of 5 mL with 4 stainless-steel balls (ø7 mm) and ground for 200 min with a Retch Mixer Mill MM 400. The frequency oscillation of 30 Hz was set for preparing the samples.

Sonication procedure: 80 mg of the ground graphite dispersed in 24 mL of sterile water was prepared for sonication. The sonication was performed at room temperature using an Ultrasonic Processor GEX750 with a flat-head tip at 450 W and 20 kHz. We performed sonication in steps of 20 s spaced out by 10 s at rest, for a total time of 5 min; at the end of the sonication, the temperature of the samples rose to 47.2 °C.

Centrifugation procedure: After sonication, the samples were transferred to pre-cleaned vials of 8 mL for centrifugation. In order to avoid the contamination of the samples, the vials were cleaned with acetone and then with hydrogen peroxide (30%) and were washed several times with sterile water in order to remove all of the organic and inorganic contaminants. The first cycle of centrifugation was carried out using a Hettich EBA21 centrifuge at 5000 rpm for 10 min to remove the fraction of the larger and thicker graphitic particles and large clusters. Then, 90% of the supernatant was separated from the sediment and it was subjected to a second centrifugation cycle at 5000 rpm for 60 min. After the second centrifugation, the supernatant (TOP) of the GNPs was separated from the sediment (BOTTOM) of the GNPs by drawing 80% of the dispersion. In this way, two types of samples of GNPs were collected after the second centrifugation. These two samples were referred to as TOP60 and BOTTOM60, according to the second centrifugation time of 60 min.

The obtained concentration w/w of TOP60 and BOTTOM60 in water was 0.29 mg/mL and 1.0 mg/mL, respectively. Since the feed GNPs dispersions, which were subjected to the second centrifugation, do not contain large GNPs or big aggregates, which are removed in the first centrifugation step, we finally obtained stable water dispersions for TOP60 and BOTTOM60.

Studies concerning GNP preparation by means of physical procedures showed that the oxidation of GNPs can occur spontaneously during milling [36,37]. By the chemical titration of the GNP dispersions, we can infer the presence of COOH groups, which will be confirmed by FTIR analysis (Section 2.4). The dispersions of TOP60 and BOTTOM60 in water were treated with HCl 0.1 M and then back-titrated with NaOH 0.06 M; phenolphthalein was used as an indicator. An equal amount of water and phenolphthalein was titrated with the same procedure and used as a reference. The higher acidity recorded for the solutions containing GNPs is consistent with the presence of acid groups, such as -COH and/or -COOH.

In order to obtain a reference sample, we followed the same sonication/centrifugation procedure illustrated above for the preparation of water dispersion containing commercial 3L and 5–7L nanoparticles.

### 3.2. Characterization of GNPs

#### 3.2.1. Dynamic Light Scattering (DLS)

The average particle sizes of GNPs (and/or of GNPs aggregates) and their distribution were estimated by dynamic light scattering (DLS) using a Zetasizer Nano ZS analyzer. The samples were diluted with distilled water to adjust their solid content to 0.05 wt% and directly placed in the cell. All of the measurements were carried out at 25 °C.

#### 3.2.2. Transmission Electron Microscopy (TEM)

Transmission electron microscopy imaging was performed using a Zeiss EM10 electron microscope. The TOP60 and BOTTOM60 GNPs in the aqueous dispersion were dropped on formvar carbon-coated grids. The grids were then air-dried at room temperature and examined. Lateral size measurements of the GNPs were performed manually using the Image Pro-Plus software (version 6.0) (Media Cybernetics, Inc., Washington, WA, USA) on randomly acquired micrographs.

#### 3.2.3. UV-Visible Extinction Spectroscopy

The UV-visible spectra were recorded using a Jasco V-570 spectrophotometer. The water dispersions of the GNPs were placed in quartz cuvettes with an optical path of 1 cm (volume of 1 mL). We acquired the UV-vis spectra in the transmission mode, using the water as a reference.

#### 3.2.4. Infrared Spectroscopy

The Infrared absorption spectra were recorded using a Thermo Nicolet NEXUS FT-IR spectrometer, equipped with a ThermoElectron-Nicolet Continuμm FT-IR microscope. The spectra of the GNPs were recorded in the double transmission (DT) mode on thin films of GNPs drop-cast from the water dispersion on reflective Al substrates (128 scan, 15X Infinity Corrected Cassegrain objective and 4 cm^−1^ resolution) and in specular reflection (SR) mode on thick and flat pressed powders of GNPs (512 scan, 15X Infinity Corrected Cassegrain objective and 4 cm^−1^ resolution). SR turned out to be very effective for thick specimens with a flat surface showing good reflectivity [33]. The SR spectra show features with the typical sigmoidal shape reminiscent of the real part of the refraction index in correspondence with the absorption (anomalous dispersion) [34,35]. To convert the reflectance spectrum into an absorption spectrum, we applied the Kramer–Koenig (KK) transformation [33], which is embedded in the software of the instrument (OMNIC 8.0).

#### 3.2.5. Raman Spectroscopy

A Jobin Yvon Labram HR800 Raman spectrometer, coupled to an Olympus BX41 microscope, with a 50X objective was used to record the Raman spectra of the GNP samples deposited on aluminum foil fixed on a glass slide. For Raman excitation, we exploited a He-Ne laser at 633 nm and diode-pumped solid-state lasers at 532 nm and 405 nm, respectively. The experiments were carried out at low power—a few hundred microwatts—of the laser beam: for each experiment, we carefully set a suitable power that prevents the degradation or photo-induced changes of the samples. The spectra were recorded as the average of five acquisitions (scan time: 120 s each) with a spectral resolution of 2 cm^−1^.

In order to rightly compare the intensity of the Raman bands, the spectra have been corrected for the instrumental response at the different wavelengths by the ICS (Intensity Correction System) routine embedded in the Raman spectrometer software LabSpec 5.58.25.

## 4. Conclusions

We have developed an effective and simple method that allows for the preparation of two different GNPs named TOP60 and BOTTOM60 in the form of stable aqueous dispersions, which could find application in the development of drug delivery platforms. The GNPs were obtained by means of the milling and sonication of synthetic graphite in the presence of air and pure water.

The high purity of the starting materials limits the chemical modifications that can take place during the GNPs preparation. The IR spectroscopy and chemical titration unveiled that the GNPs carry acid groups containing C=O, probably belonging to the carboxylic units.

The presence of these functional groups is of interest in the perspective of further functionalization aimed at improving their targeting ability and their compatibility with a biological environment. Very importantly, the Raman pattern indicates that the small graphene layers mainly maintain their integrity, suggesting that such functional groups are grafted on the edges of the graphene sheets.

The UV-vis spectroscopy indicated that the GNPs are very thin: TOP 60 exhibit an average number of stacked graphene-like layers <N> = 3.6 and for BOTTOM60 <N> = 5.7. TOP 60 contains individual, very small few-layer nanoparticles, with an average lateral size of <L> = 70 nm, which can form small aggregates in water, not showing evidence of re-stacking.

BOTTOM60 GNPs are slightly larger and more prone to form big aggregates, as observed by DLS and confirmed by the TEM images. This property could be correlated to the C=O functionalities, which are more abundant in the case of BOTTOM60.

The comparison with a commercial 3L GNP sample shows that the TOP 60 and BOTTOM60 GNPs have smaller average lateral sizes (from the TEM observations) and a more homogeneous structure (from the Raman analysis). Indeed, the Raman spectra of 3L/SC highlighted the presence of two different phases, one of the two showing a graphitic-like character, which does not appear in the case of the TOP60 and BOTTOM60 samples.

Raman spectroscopy provides powerful tools for the structural characterization of GNPs, and the IR investigation was especially useful for detecting and quantifying chemical defects.

An exceptionally wide literature concerns the Raman characterization of carbon-based materials and covers both experimental and theoretical aspects. Experimental findings and empirical spectroscopic correlations are often discussed in light of theoretical modeling. Solid-state physics and molecular spectroscopy, often supported by Quantum Mechanical simulations, provide the description of the physical phenomena which rule the Raman response of several model systems (graphite and graphene described as ideal crystals, giant graphene-like polycyclic aromatic hydrocarbons, carbon nanotubes of different diameter and chirality,…), and describe the origin of the remarkable spectroscopic features arising in the presence of structural disorder and resonance phenomena. In this rich and complex framework, we faced the discussion of the Raman spectra we collected by means of a wide experimental campaign carried out on GNPs and some reference materials. The Raman spectra analysis highlighted several peculiarities and many common structural characteristics of TOP60 and BOTTOM60 particles. Moreover, at the end of this study, we have collected a set of observations and conclusions, which we consider of general interest to researchers dealing with the study of GNPs by means of Raman spectroscopy:

(i) The Raman characterization of GNPs assemblies requires careful sampling at different points of the specimen in order to check the homogeneity of the material and to verify if the irreversible structure rearrangement has been induced by photon flux.
(ii) The use of empirical relationships to obtain structural information from Raman observables (e.g., Raman shifts, bands intensities) should be critically managed by means of a careful comparison of the spectral pattern with the Raman spectra of some reference materials.
(iii) Raman spectra deconvolution is mandatory for GNPs. However, some guidelines/protocols have to be set before the spectra processing. Indeed, the parameters extracted after the curve fitting procedure must be analyzed in a comparative way, e.g., to establish quantitative trends and/or to discuss similarities/differences among samples. For this reason, the number and type of components for the fitting must be established on the ground of physical considerations.
(iv) It is very important to verify that the trends obtained considering the parameters coming from the deconvolution (e.g., peaks frequencies or intensity ratios between individual components) are compatible with the qualitative trends which we can obtain by the direct comparison of the raw spectral data, before any mathematical processing.
(v) When interpreting the spectra, it is essential to remember that the Raman response for a given GNPs material is due to the convolution of the responses of all the different “species” which are present in the sample. Even in the case of nanoparticles with a sharp distribution of sizes, the structural disorder can make each individual particle different from the others, e.g., because of a peculiar structure of the edges or because of the different kind of layer stacking. In this respect, the commercial sample 3L/SC showed a very intriguing behavior with a Raman response resulting as the weighted sum of a graphite-like spectrum and of a spectrum presenting disorder-related features similar to that of the TOP60 GNPs.
(vi) The features described at point v. are further complicated by the occurrence of resonance phenomena: it can “select” the GNPs that better match the resonance condition because of their electronic structure. Moreover, a few peculiar vibrational modes of each individual GNP are selectively enhanced by resonance.
(vii) The study of the second-order Raman spectrum proves to be rich in information. Particles smaller than 100 nm approach a regime in between a crystalline solid (with a partial disorder) and a very large molecule. However, a widely applicable metric allowing for the extraction of quantitative structural information from two quanta transitions is still lacking. Indeed, several entangled factors determine the pattern of the second-order Raman spectrum.

## Figures and Tables

**Table 1:** Samples obtained according to different preparation steps and abbreviations adopted in the text.

Different Stages of Production	Abbreviation
After Ball Milling (60 min)	Milled60
After Balling Milling (120 min)	Milled120
After Balling Milling (180 min)	Milled180
After Balling Milling (200 min)	Milled200
After Sonication	Sonicated
Separation of supernatant after First Centrifugation (10 min)	First TOP
Separation of the sediment after First Centrifugation (10 min)	First BOTTOM
Separation of supernatant after Second Centrifugation (60 min)	TOP60
Separation of the sediment after Second Centrifugation (60 min)	BOTTOM60

**Table 2:** Average layers number <N> of GNPs samples and of commercial graphene nano-sheets according to Equation (1) (see text).

Samples	ε_550/_ε_max_	<N>
TOP60	0.35	3.6
BOTTOM60	0.54	5.7
3L/SC	0.35	3.6
5–7L/SC	0.50	6.2

**Table 3:** Ratio between the IR C=O stretching band intensity and the IR G-band intensity for different samples at different steps of the GNPs preparation. Intensity is measured as an integrated area.

Samples	A_C=O_/A_G_
HOPG	-
Rod Graphite	-
Milled200	0.51
First Bottom	0.43
BOTTOM60	0.11

**Table 4:** Parameters obtained after deconvolution of the Raman spectra (λ_exc_ = 633 nm) of the samples listed in the first column. Full Width at Half Maximum (FWHM) of the G or G_h_ line are listed in squared brackets after peaks wavenumbers (columns II and III).

Sample	Peak Position (cm^−1^)								Intensity Ratio (*)						
	D	G	G_h_	D’	D1	D2	D3	2D	ID/IG	ID’/IG	ID1/IG	ID2/IG	ID3/IG	I2D/IG	A2D/AG
HOPG	-	1582 [14]	-	-	-	-	-	2688 2647	-	-	-	-	-	0.53	1.5
ROD (i)	1336	1583 [17]	-	1621	-	1282	1494	2690 2670 2645	0.16	0.06	-	0.01	0.01	0.66	1.35
ROD (ii)	1334	1580 [24]	-	1617	1143	1282	1494	2692 2670 2649	0.45	0.11	0.02	0.04	0.02	0.61	1.24
3L/P	1336	1582 [19]	-	1619	-	-	-	2687 2662	0.21	0.05	-	-	-	0.51	0.89
3L/S	1336	1582 [19]	1590 [49]	1621	1143	1282	1494	2663	0.64	0.18	0.03	0.11	0.09	0.43	0.99
3L/SC	1334	1582 [14]	1590 [55]	1620	1143	1282	1494	2666	1.66	0.44	0.07	0.44	0.31	0.27	0.6
TOP60	1334	-	1590 [62]	1618	1143	1282	1494	2660	2.04	0.51	0.09	0.56	0.41	0.21	0.43
BOTTOM60	1332	-	1590 [65]	1616	1143	1282	1494	2659	2.3	0.51	0.13	0.56	0.42	0.27	0.52

(*) The intensity ratios are calculated as ratio between peaks heights. When several components are present (e.g., for the 2D band and for the G band in the case of 3L/S and 3L/SC) the sum of the heights of the different component is considered. This procedure can be poorly reliable and the area ratios (e.g., A2D/AG) are alternative, more suitable parameters.

**Table 5:** Intensity and area ratios of the relevant Raman band.

		ID/IG	AD/AG	ID’/IG	AD’/AG	I2D/IG	A2D/AG	ID/ID’
633 nm	TOP60	2.04	2.4	0.51	0.30	0.21	0.43	4.00
	BOTTOM 60	2.3	2.47	0.51	0.26	0.27	0.52	4.51
532 nm	TOP60	1.69	2.09	0.41	0.19	0.18	0.37	4.12
	BOTTOM 60	1.42	1.78	0.28	0.09	0.13	0.2	5.07
405 nm	TOP60	1.07	1.23	0.17	0.07	0.1	0.18	6.29
	BOTTOM 60	0.87	1.07	0.13	0.04	0.1	0.18	6.69

## Data Availability

The data presented in this study are (i) available in the article or the supplementary material or (ii) available upon request from the corresponding author.

## Supplementary Materials

The following supporting information can be downloaded at: https://www.mdpi.com/article/10.3390/molecules28020565/s1, Table S1: The hydrodynamic average diameter of GNPs in a solution of TOP60 and BOTTOM60 recorded by DLS; Figure S1: TEM images of (A) TOP60 and (B) 3L/SC GNPs; Figure S2: UV-vis extinction spectra of the GNPs samples of TOP60 and BOTTOM60 after different times; Table S2: the layer numbers of the TOP60 samples at different times after the preparation of the aqueous dispersion; Table S3. the layer numbers of BOTTOM60 samples at different times after the preparation of the aqueous dispersion; Figure S3: FTIR spectra of the samples obtained after different ball milling times of ROD graphite, characterized by specular reflection; Table S4. The ratio of C=O stretching band intensity/area to G-band intensity/area for different ball milling time in FTIR spectra; Sketch S1: Sketches of the vibrational eigenvectors (atoms displacements) associated with (a) the G and (b) the D phonons of an ideally infinite graphene sheet; Figure S4. Raman spectra of the GNP samples of different preparation processes. The Raman spectra were recorded with Green 532 nm excitation lasers with 3 mW power; Figures S5–S12: The results of bands deconvolution of the multi-wavelength Raman spectra of the samples analyzed; Tables S5–S13: the parameters were obtained by means of bands deconvolution of the multi-wavelength Raman spectra of the samples analyzed; Table S14: (a) Description and number of Loretzian/Voigt components adopted in the fit of the Raman spectra of some selected samples. (b) Curve type selected for the individual components; Figure S13. The Raman spectra of the GNP samples of TOP60 and BOTTOM60 excited by red (633 nm) laser at the different power values in the same point; Figure S14. The Raman spectra of the GNP samples of TOP60 and BOTTOM60 were excited using different laser wavelengths. References [71],[72],[73] are cited in the supplementary materials.
