# Supplementary material for: Development of Tailored Graphene Nanoparticles: Preparation, Sorting and Structure Assessment by Complementary Techniques
Source: Molecules. 2023 Jan 5;28(2):565. doi: 10.3390/molecules28020565 (PMC9865347; doi:10.3390/molecules28020565)
Supplement: Supplementary file 1 [file molecules-28-00565-s001.zip › molecules-2054751-supplementary.pdf]

# Supporting Information

# Development of tailored graphene nanoparticles: preparation, sorting and structure assessment by complementary techniques

Kaiyue Hu[1], Luigi Brambilla[1], Patrizia Sartori[2], Claudia Moscheni[3], Cristiana Perrotta[3], Lucia Zema[4*], Chiara Bertarelli[1,5], Chiara Castiglioni[1]

[1] Dipartimento di Chimica, Materiali e Ingegneria Chimica Giulio Natta, Politecnico di Milano, piazza Leonardo da Vinci 32, 20133 Milano (Italy)

[2] Dipartimento di Scienze Biomediche per la Salute, Facoltà di Medicina e Chirurgia, Università degli Studi di Milano, Via G. Colombo 71, 20133 Milano (Italy)

[3] Dipartimento di Scienze Biomediche e Cliniche, Università degli Studi di Milano, via Gian Battista Grassi 74, 20157 Milano (Italy)

[4] Sezione di Tecnologia e Legislazione Farmaceutiche "M. E. Sangalli", Dipartimento di Scienze Farmaceutiche, Università degli Studi di Milano, Via Giuseppe Colombo,71 20133 Milano (Italy)

[5] Center for Nano Science and Technology @PoliMi, Istituto Italiano di Tecnologia, via Pascoli 70/3, 20133 Milano (Italy)

## 1. DLS data

Table S1. The hydrodynamic average diameter of GNPs in solution of TOP60 and BOTTOM60 recorded by DLS.

| Size/Samples | TOP60 | BOTTOM60 |
|---|---|---|
| Z-Avg | 107.0 nm | 192.2 nm |

## 2. TEM

TEM was used to analyze the commercial 3L/SC GNPs/nanoparticles sample. Unlike the TOP60 and BOTTOM60 GNPs, individual 3L/SC graphene nano-sheets show a polygonal profile with straight and regular edges.

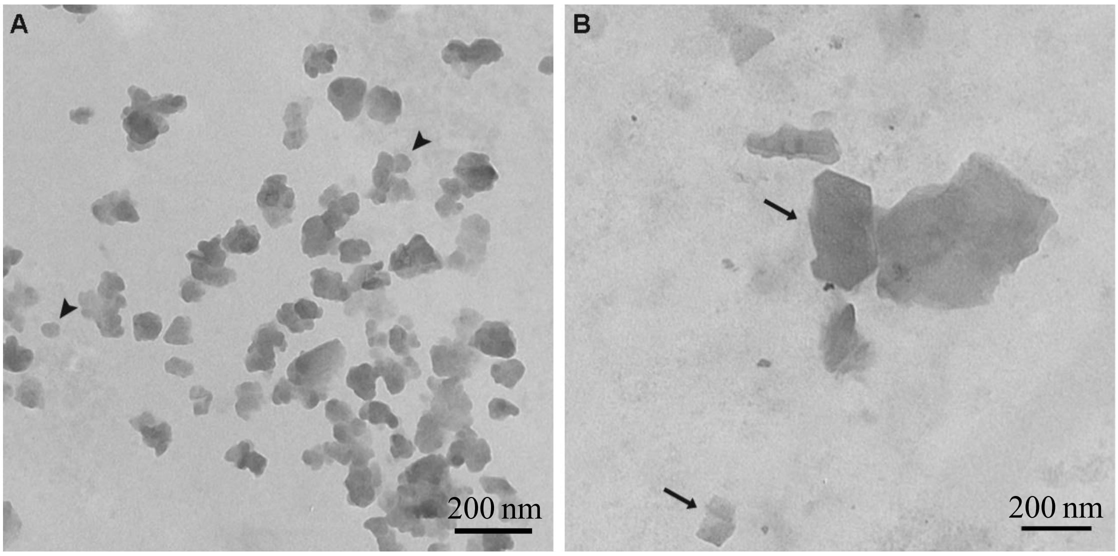


Figure S1. TEM images of (A) TOP60 and (B) 3L/SC GNPs. TOP60 nanoparticles exhibit an irregular shape with rounded edges (arrowheads) while the 3L/SC flakes show a polygonal shape with straight edges (arrows). Scale bar: 200 nm.

## 3. UV-vis spectra

*Time evolution*

Figure S2 shows that at different times (1-165 days) there are almost no changes in

the UV-vis absorption spectra of TOP60 and the estimated number of layers is close to 3 - 4 layers. Instead, for BOTTOM60, as time increases, the spectra change showing a broader peak with increasing time, probably due to GNPs aggregation phenomena. Therefore, TOP60 nanoparticles result to be stable in aqueous solution. BOTTOM60 shows phenomena of aggregation after 45 days. However there is no evidence of re-stacking (the average number of layers does not change with time). The number of layers of nanoparticles at different times, according to the equation (S1), are shown in Table S2 and Table S3.

$$< N >= 13.7 \times {}^{\varepsilon_{550}}/_{\varepsilon_{max}} - 1.2 \quad \text{(eq.S1)}$$

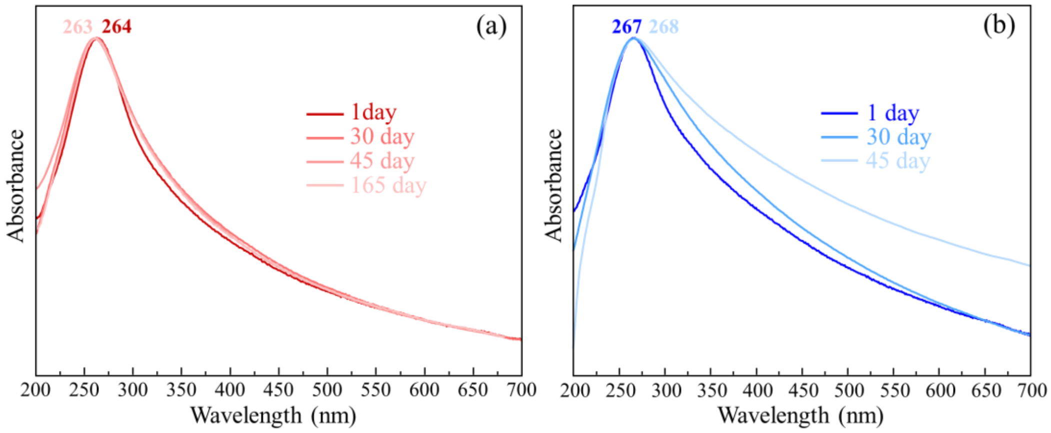


Figure S2. UV-vis extinction spectra of the GNPs samples of TOP60, BOTTOM60 at after different times. (a) TOP60, after 1, 30, 45, and 165 days for; (b) BOTTOM60, after 1, 30, and 45 days.

Table S2. The layer numbers of TOP60 samples at different times after the preparation of the aqueous dispersion, according to eqn (S1).

| **Samples** | $\varepsilon_{550}/\varepsilon_{max}$ | **<N>** |
|---|---|---|
| TOP60-1 | 0.35 | 3.6 |
| TOP60-30 | 0.37 | 3.9 |
| TOP60-45 | 0.38 | 3.9 |
| TOP60-165 | 0.37 | 3.9 |

Table S3. The layer numbers of BOTTOM60 samples at different times, after the preparation of the aqueous dispersion, according to eqn (S1).

| **Samples** | $\varepsilon_{550}/\varepsilon_{max}$ | **<N>** |
|---|---|---|
| BOTTOM60-1 | 0.54 | 6.2 |
| BOTTOM60-30 | 0.59 | 6.9 |
| BOTTOM60-45 | 0.60 | 7 |

## 4. IR spectra

*Infrared spectra after ball milling*

In Figure S3, we show the specular reflection (SR) IR spectra of GNPs samples recorded after **different milling time.** Figure S3 (a) displays the raw SR experimental spectra; after Kramers-Kronig conversion (Figure S3 (b)) a clear C=O stretching band near 1720 $cm^{-1}$ appears in the absorption spectrum, which is better appreciated after baseline correction Figure S3 (c)). Nanoparticles are subjected to strong mechanical shearing force during ball milling, therefore exogenous doping defects (including various functional groups and heteroatoms, etc.) and/or self-doping defects (including vacancies, Stone-Wales defects, edges, dislocations, grain boundaries, etc.) would take place during the milling. In Table S4, the extent of C=O functionalization is estimated by measuring the ratio of peak heights of the C=O stretching band (C=O band intensity) and the peak height of the G-band for different ball milling time in FTIR spectra. An alternative estimation is given by the ratio of the integrated areas of the C=O stretching and G-band, which should be roughly linearly proportional to the concentration ratios between C=O and CC bonds.

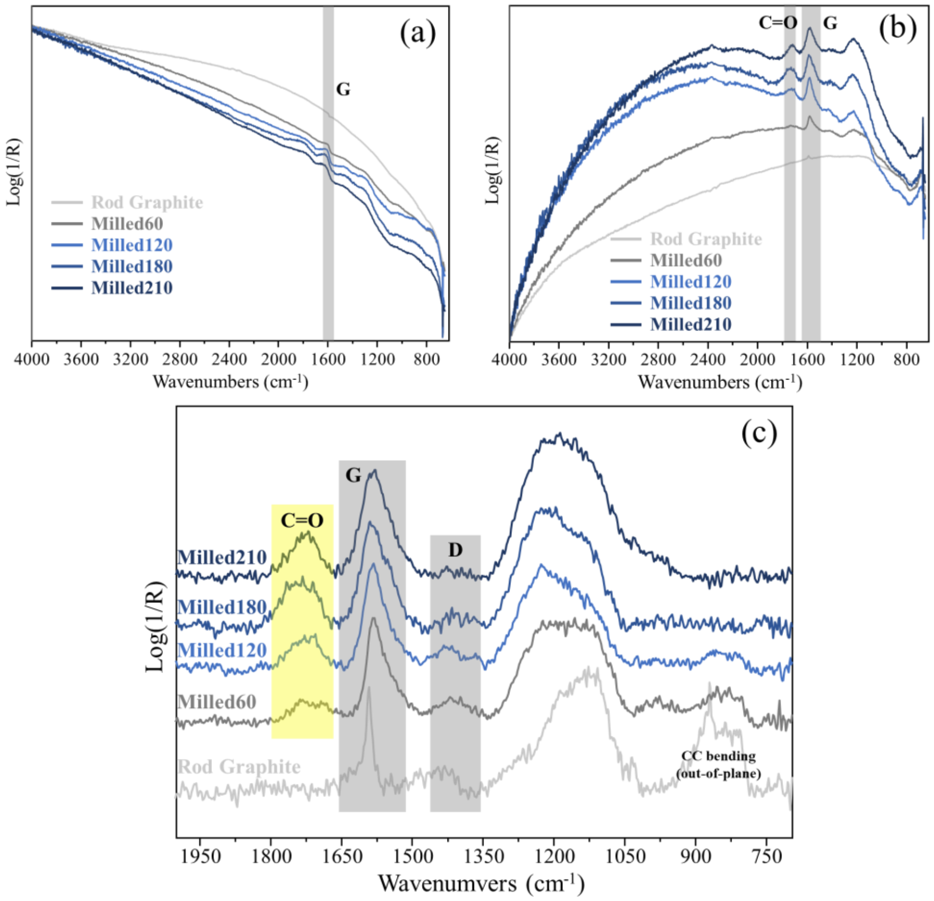


Figure S3. FTIR spectra of the samples obtained after different ball milling time of ROD graphite, characterized by specular reflection. (a) Raw experimental spectra; (b) Experimental spectra after Kramers-Kronig conversion; And (c) Experimental spectra after auto-baseline calibration.

Table S4. The ratio of C=O stretching band intensity/area to G-band intensity/area for different ball milling time in FTIR spectra.

| Samples | Intensity (C=O) [arb units] | Intensity (G) [arb units] | $I_{C=O}/I_G$ | Area (C=O) [arb units] | Area (G) [arb units] | $A_{C=O}/A_G$ |
|---|---|---|---|---|---|---|
| Milled60 | 0.01 | 0.07 | 0.14 | 0.93 | 3.33 | 0.28 |
| Milled120 | 0.03 | 0.10 | 0.30 | 2.54 | 6.02 | 0.42 |
| Milled180 | 0.04 | 0.09 | 0.44 | 3.98 | 7.18 | 0.55 |
| Milled210 | 0.03 | 0.07 | 0.43 | 2.66 | 5.76 | 0.46 |

## 5. Raman spectra

| | |
|---|---|
| 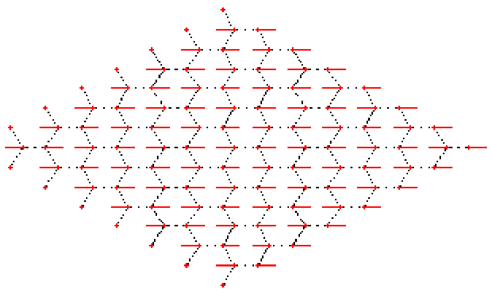 | 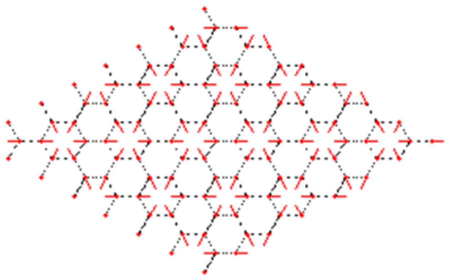 |
| a. Graphite $E_{2g}$ phonon<br>(point Γ of BZ) | b. Graphite $A'_1$ phonon<br>(K point of BZ) |

Sketch S1 - Sketches of the vibrational eigenvectors (atoms displacements) associated to: (a) the G (**q**= **Γ**) and (b) the D (**q**= **K**) phonons of an ideally infinite graphene sheet.

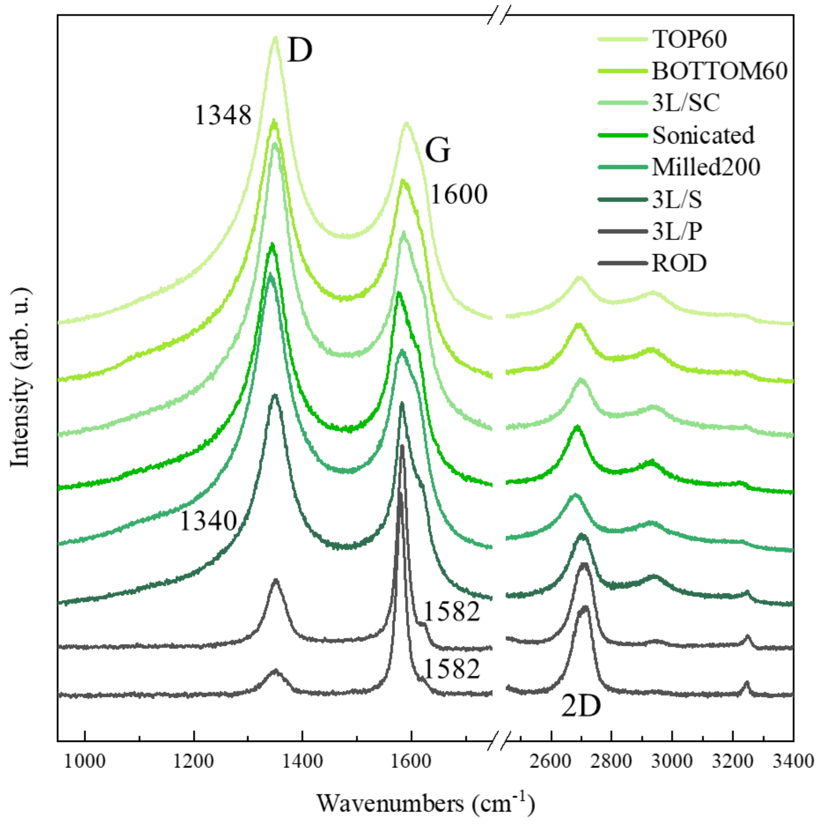


Figure S4. Raman spectra of the GNP samples of different preparation processes. Raman spectra were recorded with Green 532 nm excitation lasers with 3 mW power.

*Deconvolution of the Raman spectra, by means of curve fitting procedure.*

An accurate analysis of the Raman pattern takes into account band shapes, and is grounded on curve fitting procedures which allows identifying contributions from different phases or from different regions of the same particle (ordered staking of large sheets, confined domains with edges or small particles, very disorderd regions,….).

Figures S5-S12 illustrate the result of the curve fitting of the Raman spectra of several reference materials (HOPG, ROD) and GNPs (3L, TOP60, BOTTOM60), recorded at different excitation wavelengths. In Tables S5-S13 several relevant parameters of the individual bands components (from curve fitting) are listed.

The intensity ratios are calculated as ratio between peaks heights, which are labelled by the letter "I" followed by the indication of the Raman line, e.g. ID/IG. When several components are present (e.g. for the 2D band of the most samples and for the G band in the case of 3L/S and 3L/SC, showing G and Gh components) the sum of the heights of the different band component is considered, for the calculation of the ratios. This procedure is sometimes poorly reliable and the area ratios (e.g. A2D/AG) are alternative, more suitable parameters for sake of comparison.

While the integrated band area should be considered the more reliable parameter, because it corresponds to the Raman Cross section associated to a given transition, usually the increasing/decreasing trends of height ratio and area ratio are consistent. This feature suggests that the simpler analysis of the bands height ratios should be exploited, especially for a quick preliminary diagnosis, thus avoiding band deconvolution. However, when several components are relevant for a good curve fitting, the heigh of a peak is affected by the different components and the height ratio becomes poorly reliable. In this case a careful deconvolution of the spectra and the use of area ratios become mandatory.

Interestingly, probing ROD graphite in different points shows that the sample in not structurally homogeneous on the micrometer scale, and consists of an assembly of graphite domains characterized by different amount of disorder. For instance the Raman

spectra reported in Figure S6 and S7 - ROD(i) and ROD(ii) spectra – show a rather different ID/IG value, from 0.16 to 0.45.

For this reason, we have included the data obtained from the curve fitting of ROD(i) and ROD(ii) spectra.

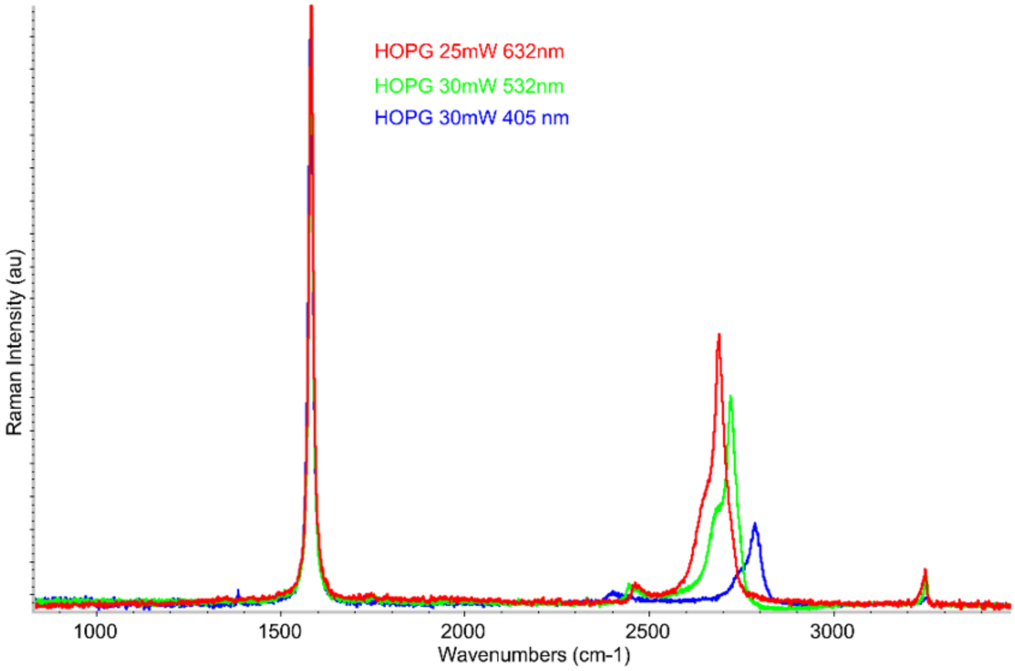


Figure S5(a) Raman spectra of HOPG at different excting laser wavelengths.

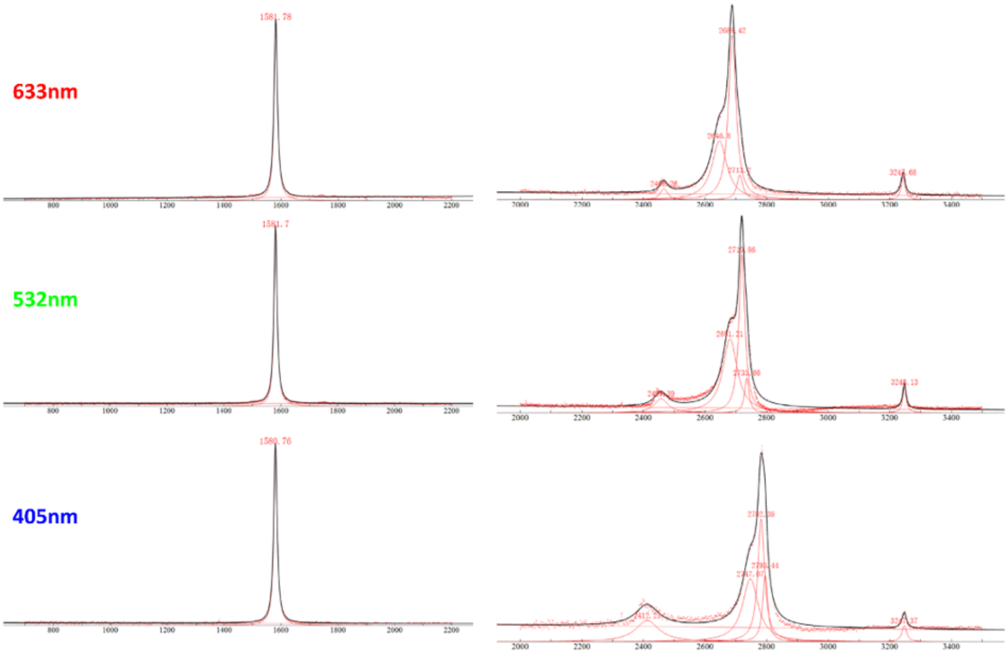


Figure S5(b) Raman spectra of HOPG at different excting laser wavelengths: fitting results.

Table S5. Fitting parameters of Raman spectra of HOPG at different exciting laser wavelengths and relevant ratios of peaks intensities.

| 633 HOPG – first order | | | | | |
|---|---|---|---|---|---|
| PeakType | | Center | Height | Area | FWHM |
| Lorentzian | G | 1582 | 0.98 | 22.09 | 14.29 |

| 633 HOPG - 2D region | | | | |
|---|---|---|---|---|
| PeakType | Center | Height | Area | FWHM |
| Lorentzian | 2466 | 0.03 | 1.21 | 29.32 |
| Lorentzian | 2647 | 0.14 | 12.75 | 58.86 |
| Lorentzian | 2688 | 0.38 | 17.99 | 29.77 |
| Lorentzian | 2713 | 0.06 | 2.19 | 23.94 |
| Lorentzian | 3244 | 0.05 | 1.31 | 15.70 |

633nm

| I2D/IG | 0.53 | A2D/AG | 1.50 |
|---|---|---|---|

| 532 HOPG – first order | | | | | |
|---|---|---|---|---|---|
| PeakType | | Center | Height | Area | FWHM |
| Lorentzian | G | 1582 | 0.98 | 20.29 | 13.18 |

| 532 HOPG - 2D region | | | | |
|---|---|---|---|---|
| PeakType | Center | Height | Area | FWHM |
| Lorentzian | 2457 | 0.03 | 2.14 | 52.92 |
| Lorentzian | 2681 | 0.13 | 12.02 | 57.15 |
| Lorentzian | 2720 | 0.29 | 10.45 | 23.21 |
| Lorentzian | 2736 | 0.06 | 1.68 | 16.87 |
| Lorentzian | 3248 | 0.05 | 1.01 | 13.76 |

532nm

| I2D/IG | 0.44 | A2D/AG | 1.19 |
|---|---|---|---|

| 405 HOPG – first order | | | | | |
|---|---|---|---|---|---|
| PeakType | | Center | Height | Area | FWHM |
| Lorentzian | G | 1581 | 0.99 | 21.95 | 14.11 |

| 405 HOPG – 2D region | | | | |
|---|---|---|---|---|
| PeakType | Center | Height | Area | FWHM |
| Lorentzian | 2412 | 0.02 | 2.21 | 91.11 |
| Lorentzian | 2747 | 0.05 | 4.40 | 61.36 |
| Lorentzian | 2782 | 0.09 | 3.69 | 26.41 |
| Lorentzian | 2795 | 0.05 | 1.49 | 19.72 |
| Lorentzian | 3247 | 0.01 | 0.34 | 18.71 |

405nm

| I2D/IG | 0.16 | A2D/AG | 0.44 |
|---|---|---|---|

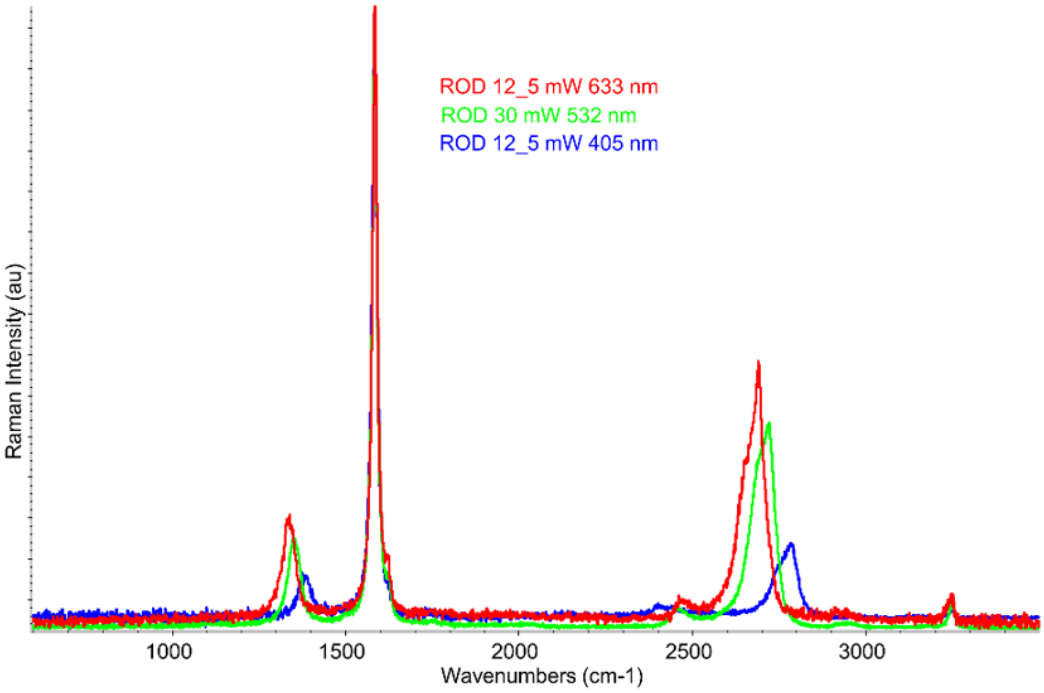


Figure S6 (a) Raman spectra of ROD (i) at different exciting laser wavelengths.

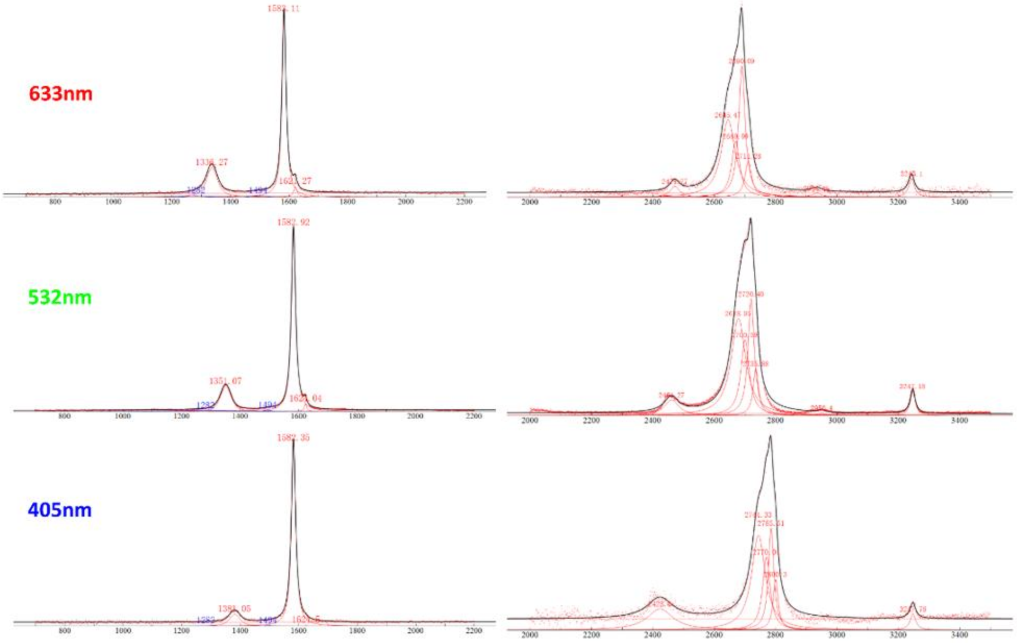


Figure S6 (b) Raman spectra of ROD (i) at different exciting laser wavelengths: fitting results.

Table S6. Fitting parameters of Raman spectra of ROD(i) at different exciting laser wavelengths and relevant ratios of peaks intensities.

| 633 ROD G (i) – first order | | | | | |
|---|---|---|---|---|---|
| PeakType | | Center | Height | Area | FWHM |
| Lorentzian | D2 | 1282 | 0.01 | 0.64 | 63.21 |
| Voigt | D | 1336 | 0.16 | 9.81 | 11.17 |
| Gaussian | D3 | 1494 | 0.01 | 0.39 | 58.13 |
| Lorentzian | G | 1583 | 0.98 | 26.00 | 16.82 |
| Lorentzian | D' | 1621 | 0.06 | 1.20 | 13.68 |

| 633 ROD G (i) 2D region | | | | |
|---|---|---|---|---|
| PeakType | Center | Height | Area | FWHM |
| Lorentzian | 2472 | 0.02 | 1.37 | 37.17 |
| Lorentzian | 2645 | 0.17 | 14.60 | 55.46 |
| Lorentzian | 2670 | 0.12 | 6.05 | 31.70 |
| Lorentzian | 2690 | 0.28 | 11.36 | 25.40 |
| Lorentzian | 2711 | 0.08 | 3.20 | 25.92 |
| Lorentzian (G+D) | 2934 | 0.01 | 0.55 | 39.97 |
| Lorentzian | 3243 | 0.04 | 1.41 | 22.33 |

| 532 ROD G - (i) – first order | | | | | |
|---|---|---|---|---|---|
| PeakType | | Center | Height | Area | FWHM |
| Lorentzian | D2 | 1282 | 0.00 | 2.51 | 392.62 |
| Voigt | D | 1351 | 0.13 | 7.70 | 43.79 |
| Gaussian | D3 | 1494 | 0.00 | 0.18 | 35.11 |
| Lorentzian | G | 1583 | 0.99 | 27.15 | 17.54 |
| Lorentzian | D' | 1624 | 0.04 | 0.64 | 9.77 |

| 532 ROD G - (i) – 2D region | | | | |
|---|---|---|---|---|
| PeakType | Center | Height | Area | FWHM |
| Lorentzian | 2461 | 0.03 | 2.35 | 56.33 |
| Lorentzian | 2679 | 0.16 | 14.78 | 57.10 |
| Lorentzian | 2701 | 0.13 | 6.73 | 33.42 |
| Lorentzian | 2720 | 0.20 | 7.57 | 24.31 |
| Lorentzian | 2736 | 0.08 | 2.67 | 21.31 |
| Lorentzian (G+D) | 2950 | 0.00 | 0.14 | 20.37 |
| Lorentzian | 3247 | 0.04 | 1.26 | 19.31 |

| 405 ROD G – first order | | | | | |
|---|---|---|---|---|---|
| PeakType | | Center | Height | Area | FWHM |
| Lorentzian | D2 | 1282 | 0.00 | 0.18 | 93.75 |
| Voigt | D | 1381 | 0.06 | 3.82 | 43.54 |
| Gaussian | D3 | 1494 | 0.00 | 0.17 | 65.87 |
| Lorentzian | G | 1582 | 0.98 | 27.28 | 17.69 |
| Lorentzian | D' | 1625 | 0.01 | 0.03 | 3.17 |

| 405 ROD G – 2D region | | | | |
|---|---|---|---|---|
| PeakType | Center | Height | Area | FWHM |
| Lorentzian | 2424 | 0.01 | 2.17 | 102.56 |
| Lorentzian | 2744 | 0.06 | 5.18 | 54.45 |
| Lorentzian | 2770 | 0.05 | 2.17 | 29.52 |
| Lorentzian | 2786 | 0.07 | 2.21 | 21.54 |
| Lorentzian | 2800 | 0.03 | 1.02 | 20.09 |
| Lorentzian | 3248 | 0.01 | 0.36 | 22.25 |

633nm

| | | | |
|---|---|---|---|
| ID/IG | 0.16 | AD/AG | 0.38 |
| ID'/IG | 0.06 | AD'/AG | 0.05 |
| I2D/IG | 0.66 | A2D/AG | 1.35 |
| | | | |
| ID2/IG | 0.01 | AD2/AG | 0.02 |
| ID3/IG | 0.01 | AD3/AG | 0.01 |

532nm

| | | | |
|---|---|---|---|
| ID/IG | 0.14 | AD/AG | 0.28 |
| ID'/IG | 0.04 | AD'/AG | 0.02 |
| I2D/IG | 0.58 | A2D/AG | 1.17 |
| | | | |
| ID2/IG | 0.00 | AD2/AG | 0.09 |
| ID3/IG | 0.00 | AD3/AG | 0.01 |

405nm

| | | | |
|---|---|---|---|
| ID/IG | 0.06 | AD/AG | 0.14 |
| ID'/IG | 0.01 | AD'/AG | 0.00 |
| I2D/IG | 0.21 | A2D/AG | 0.39 |
| | | | |
| ID2/IG | 0.00 | AD2/AG | 0.01 |
| ID3/IG | 0.00 | AD3/AG | 0.01 |

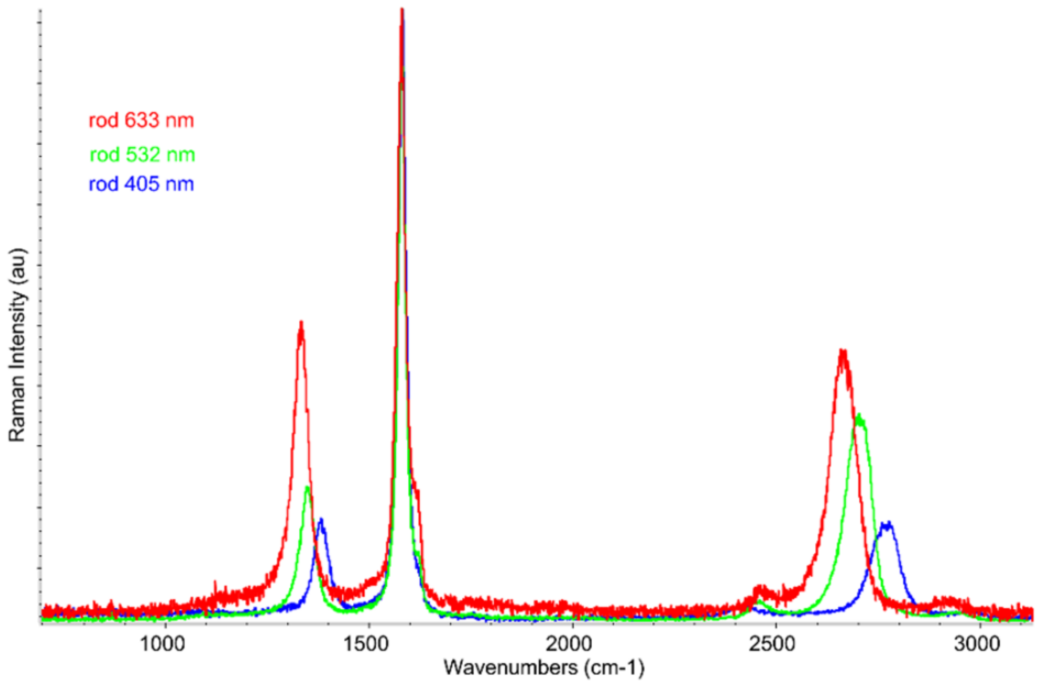


Figure S7(a) Raman spectra of ROD (ii) at different exciting laser wavelengths.

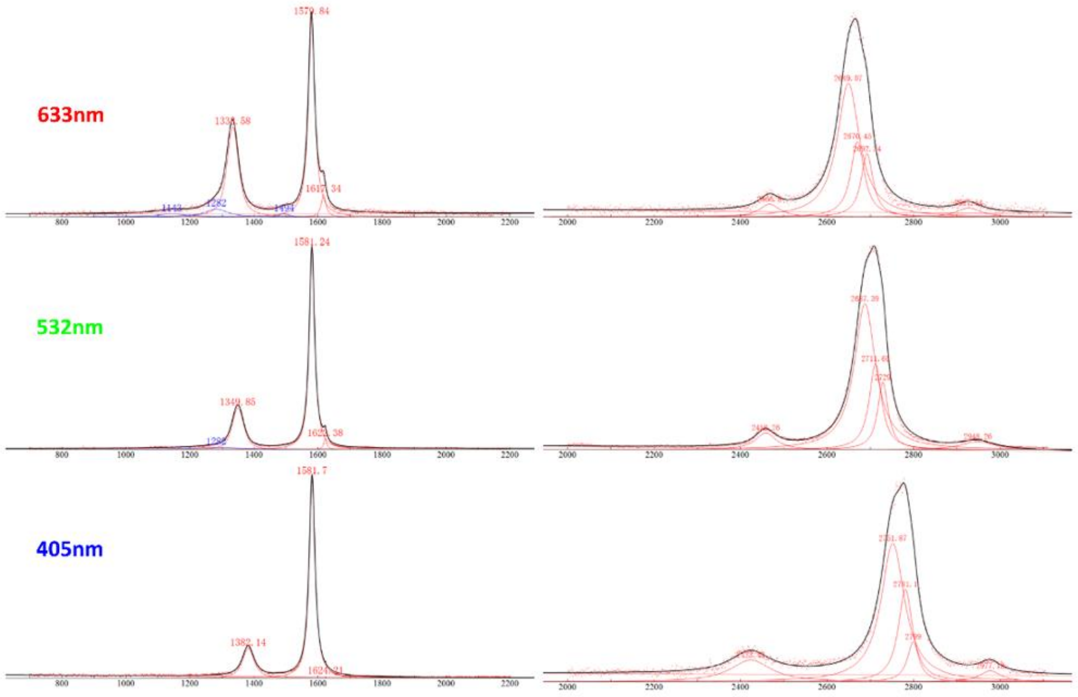


Figure S7(b) Raman spectra of ROD (ii) at different exciting laser wavelengths: fitting results.

Table S7. Fitting parameters of Raman spectra of ROD(ii) at different exciting laser wavelengths and relevant of peaks intensities.

| 633 ROD G (ii) – first order | | | | | |
|---|---|---|---|---|---|
| PeakType | | Center | Height | Area | FWHM |
| Lorentzian | D1 | 1143 | 0.02 | 2.95 | 122.90 |
| Lorentzian | D2 | 1282 | 0.04 | 5.33 | 86.82 |
| Voigt | D | 1334 | 0.44 | 26.79 | 43.12 |
| Gaussian | D3 | 1494 | 0.01 | 0.68 | 43.21 |
| Lorentzian | G | 1580 | 0.98 | 37.36 | 24.22 |
| Lorentzian | D' | 1617 | 0.11 | 3.05 | 17.65 |
| **532 ROD G (ii) – first order** | | | | | |
| PeakType | | Center | Height | Area | FWHM |
| Lorentzian | D2 | 1282 | 0.01 | 3.40 | 200.78 |
| Voigt | D | 1350 | 0.20 | 11.75 | 42.93 |
| Lorentzian | G | 1581 | 0.99 | 31.72 | 20.40 |
| Lorentzian | D' | 1622 | 0.05 | 0.96 | 11.53 |
| **405 ROD G (ii) – first order** | | | | | |
| PeakType | | Center | Height | Area | FWHM |
| Voigt | D | 1382 | 0.15 | 9.55 | 43.12 |
| Lorentzian | G | 1582 | 1.02 | 35.42 | 22.22 |
| Lorentzian | D' | 1624 | 0.01 | 0.02 | 1.26 |

| 633 ROD G (ii) – 2D region | | | | |
|---|---|---|---|---|
| PeakType | Center | Height | Area | FWHM |
| Lorentzian | 2467 | 0.03 | 2.57 | 57.44 |
| Lorentzian | 2649 | 0.30 | 28.90 | 62.32 |
| Lorentzian | 2670 | 0.17 | 9.96 | 38.06 |
| Lorentzian | 2692 | 0.14 | 7.36 | 33.49 |
| Lorentzian (G+D) | 2928 | 0.02 | 2.19 | 70.05 |
| **532 ROD G (ii) – 2D region** | | | | |
| PeakType | Center | Height | Area | FWHM |
| Lorentzian | 2458 | 0.03 | 2.49 | 57.24 |
| Lorentzian | 2687 | 0.24 | 22.50 | 59.41 |
| Lorentzian | 2712 | 0.14 | 7.69 | 34.51 |
| Lorentzian | 2729 | 0.11 | 5.15 | 29.45 |
| Lorentzian (G+D) | 2948 | 0.01 | 1.92 | 91.32 |
| **405 ROD G (ii) – 2D region** | | | | |
| PeakType | Center | Height | Area | FWHM |
| Lorentzian | 2423 | 0.02 | 3.16 | 113.09 |
| Lorentzian | 2752 | 0.11 | 11.68 | 66.32 |
| Lorentzian | 2781 | 0.07 | 4.69 | 39.85 |
| Lorentzian | 2799 | 0.03 | 1.65 | 32.40 |
| Lorentzian (G+D) | 2977 | 0.01 | 0.86 | 61.10 |

| 633nm | | | |
|---|---|---|---|
| ID/IG | 0.45 | AD/AG | 0.72 |
| ID'/IG | 0.11 | AD'/AG | 0.08 |
| I2D/IG | 0.61 | A2D/AG | 1.24 |
| | | | |
| ID1/IG | 0.02 | AD1/AG | 0.08 |
| ID2/IG | 0.04 | AD2/AG | 0.14 |
| ID3/IG | 0.02 | AD3/AG | 0.02 |
| **532nm** | | | |
| ID/IG | 0.21 | AD/AG | 0.37 |
| ID'/IG | 0.05 | AD'/AG | 0.03 |
| I2D/IG | 0.50 | A2D/AG | 1.11 |
| | | | |
| ID2/IG | 0.01 | AD2/AG | 0.11 |
| **405nm** | | | |
| ID/IG | 0.15 | AD/AG | 0.27 |
| ID'/IG | 0.01 | AD'/AG | 0.00 |
| I2D/IG | 0.22 | A2D/AG | 0.51 |

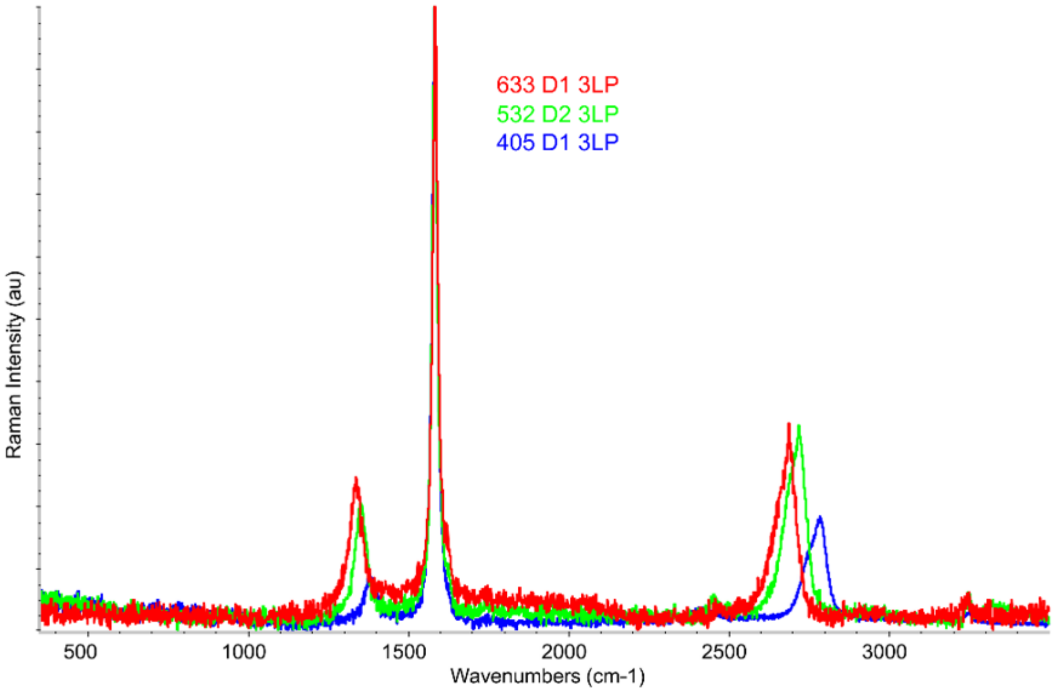


Figure S8(a) Raman spectra of 3L/P at different exciting laser wavelengths.

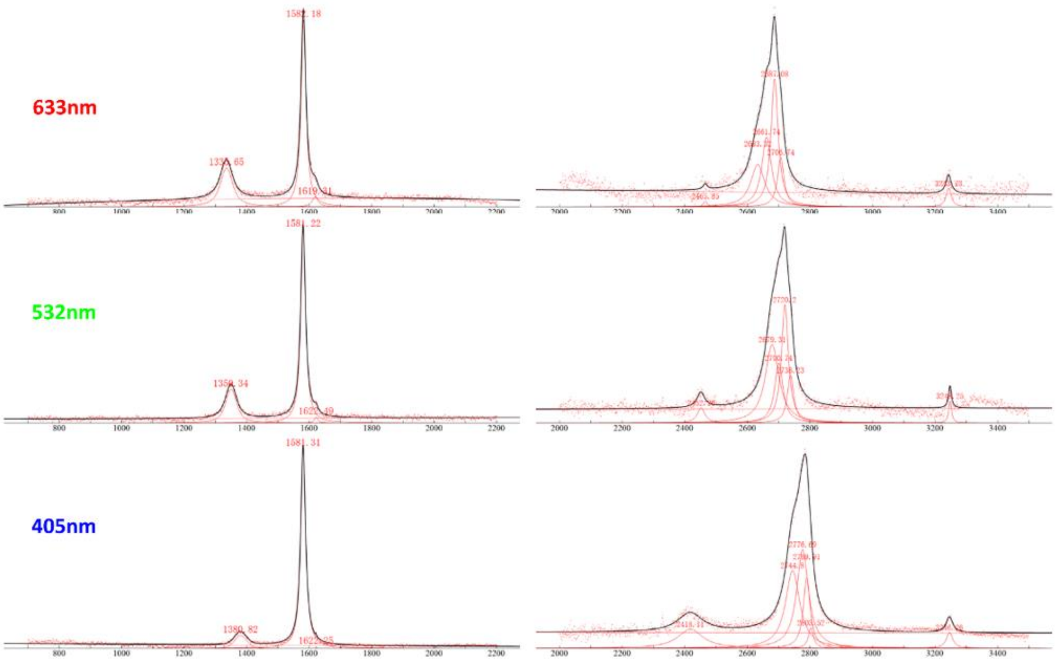


Figure S8(b) Raman spectra of 3L/P at different exciting laser wavelengths: fitting results.

Table S8. Fitting parameters of Raman spectra of 3L/P at different exciting laser wavelengths and relevant of peaks intensities.

| 633 3LP – first order | | | | | |
|---|---|---|---|---|---|
| PeakType | | Center | Height | Area | FWHM |
| Voigt | D | 1336 | 0.20 | 13.84 | 46.99 |
| Lorentzian | G | 1582 | 0.94 | 28.27 | 19.09 |
| Lorentzian | D' | 1619 | 0.05 | 1.89 | 23.51 |

| 633 3LP –2D region | | | | |
|---|---|---|---|---|
| PeakType | Center | Height | Area | FWHM |
| Lorentzian | 2466 | 0.01 | 0.23 | 14.79 |
| Lorentzian | 2633 | 0.07 | 6.02 | 54.08 |
| Lorentzian | 2662 | 0.12 | 6.84 | 37.24 |
| Lorentzian | 2687 | 0.21 | 9.16 | 27.27 |
| Lorentzian | 2707 | 0.08 | 3.28 | 25.23 |
| Lorentzian | 3244 | 0.03 | 0.98 | 19.82 |

| 633nm | | | |
|---|---|---|---|
| ID/IG | 0.21 | AD/AG | 0.49 |
| ID'/IG | 0.05 | AD'/AG | 0.07 |
| I2D/IG | 0.51 | A2D/AG | 0.89 |

| 532 3LP – first order | | | | | |
|---|---|---|---|---|---|
| PeakType | | Center | Height | Area | FWHM |
| Voigt | D | 1350 | 0.17 | 10.21 | 42.58 |
| Lorentzian | G | 1581 | 0.98 | 28.60 | 18.65 |
| Lorentzian | D' | 1622 | 0.03 | 0.63 | 13.33 |

| 532 3LP – 2D region | | | | |
|---|---|---|---|---|
| PeakType | Center | Height | Area | FWHM |
| Lorentzian | 2452 | 0.03 | 1.19 | 29.88 |
| Lorentzian | 2679 | 0.13 | 10.84 | 53.09 |
| Lorentzian | 2701 | 0.10 | 5.06 | 32.36 |
| Lorentzian | 2720 | 0.20 | 8.22 | 26.67 |
| Lorentzian | 2738 | 0.08 | 3.08 | 24.68 |
| Lorentzian | 3248 | 0.04 | 0.63 | 10.90 |

| 532nm | | | |
|---|---|---|---|
| ID/IG | 0.17 | AD/AG | 0.36 |
| ID'/IG | 0.03 | AD'/AG | 0.02 |
| I2D/IG | 0.52 | A2D/AG | 0.95 |

| 405 3LP – first order | | | | | |
|---|---|---|---|---|---|
| PeakType | | Center | Height | Area | FWHM |
| Voigt | D | 1381 | 0.06 | 3.47 | 45.76 |
| Lorentzian | G | 1581 | 1.01 | 29.62 | 18.75 |
| Lorentzian | D' | 1622 | 0.01 | 0.03 | 1.49 |

| 405 3LP – 2D region | | | | |
|---|---|---|---|---|
| # PeakType | Center | Height | Area | FWHM |
| Lorentzian | 2418 | 0.02 | 2.70 | 97.26 |
| Lorentzian | 2745 | 0.07 | 6.21 | 54.98 |
| Lorentzian | 2777 | 0.09 | 6.56 | 45.61 |
| Lorentzian | 2790 | 0.07 | 2.71 | 26.42 |
| Lorentzian | 2804 | 0.02 | 0.59 | 19.75 |
| Lorentzian | 3246 | 0.01 | 0.57 | 24.88 |

| 405nm | | | |
|---|---|---|---|
| ID/IG | 0.06 | AD/AG | 0.12 |
| ID'/IG | 0.01 | AD'/AG | 0.001 |
| I2D/IG | 0.25 | A2D/AG | 0.54 |

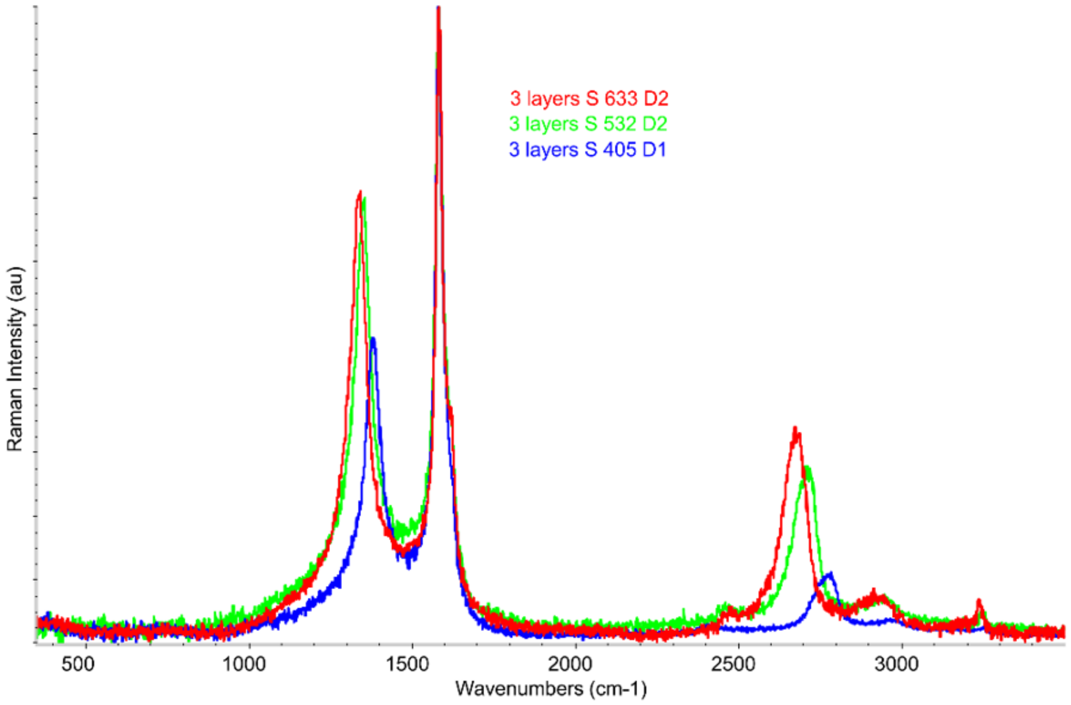


Figure S9(a) Raman spectra of 3L/S at different exciting laser wavelengths.

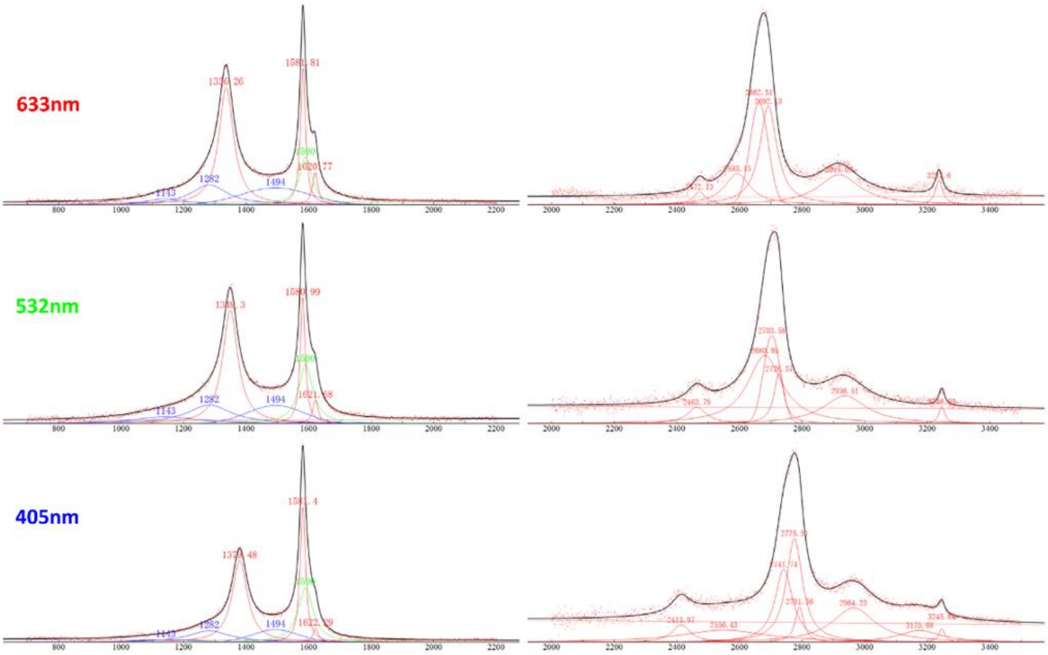


Figure S9(b) Raman spectra of 3L/S at different exciting laser wavelengths: fitting results.

Table S9. Fitting parameters of Raman spectra of 3L/S at different exciting laser wavelengths and relevant of peaks intensities.

| 633 3LS – first order | | | | | |
|---|---|---|---|---|---|
| PeakType | | Center | Height | Area | FWHM |
| Lorentzian | D1 | 1143 | 0.03 | 7.00 | 144.63 |
| Lorentzian | D2 | 1282 | 0.10 | 22.13 | 140.55 |
| Voigt | D | 1336 | 0.59 | 52.91 | 57.12 |
| Gaussian | D3 | 1494 | 0.09 | 23.37 | 258.21 |
| Lorentzian | G | 1582 | 0.69 | 20.50 | 19.00 |
| Lorentzian | Gh | 1590 | 0.24 | 18.39 | 49.10 |
| Lorentzian | D' | 1621 | 0.16 | 5.29 | 20.77 |

| 2D region | | | | |
|---|---|---|---|---|
| PeakType | Center | Height | Area | FWHM |
| Lorentzian | 2472 | 0.02 | 1.63 | 46.58 |
| Lorentzian | 2593 | 0.05 | 8.35 | 99.70 |
| Voigt | 2663 | 0.18 | 12.73 | 68.20 |
| Lorentzian | 2692 | 0.17 | 17.62 | 66.40 |
| Lorentzian (G+D) | 2918 | 0.05 | 13.16 | 162.20 |
| Lorentzian | 3238 | 0.04 | 1.82 | 27.63 |

| 532 3LS – first order | | | | | |
|---|---|---|---|---|---|
| PeakType | | Center | Height | Area | FWHM |
| Lorentzian | D1 | 1143 | 0.03 | 13.77 | 255.11 |
| Lorentzian | D2 | 1282 | 0.09 | 24.96 | 171.12 |
| Voigt | D | 1349 | 0.57 | 52.61 | 59.04 |
| Gaussian | D3 | 1494 | 0.09 | 20.55 | 211.82 |
| Lorentzian | G | 1581 | 0.63 | 18.84 | 18.95 |
| Lorentzian | Gh | 1590 | 0.30 | 25.83 | 55.14 |
| Lorentzian | D' | 1622 | 0.12 | 4.01 | 21.78 |

| 532 3LS – 2D region | | | | |
|---|---|---|---|---|
| PeakType | Center | Height | Area | FWHM |
| Lorentzian | 2464 | 0.02 | 2.69 | 74.31 |
| Lorentzian | 2681 | 0.10 | 20.06 | 134.18 |
| Voigt | 2704 | 0.12 | 8.32 | 63.42 |
| Lorentzian | 2726 | 0.07 | 4.24 | 38.42 |
| Lorentzian (G+D) | 2937 | 0.04 | 10.32 | 168.53 |
| Lorentzian | 3247 | 0.02 | 0.86 | 22.87 |

| 405 3LS – first order | | | | | |
|---|---|---|---|---|---|
| PeakType | | Center | Height | Area | FWHM |
| Lorentzian | D1 | 1143 | 0.01 | 4.90 | 242.84 |
| Lorentzian | D2 | 1282 | 0.06 | 14.34 | 159.40 |
| Voigt | D | 1379 | 0.41 | 38.05 | 59.06 |
| Gaussian | D3 | 1494 | 0.06 | 11.59 | 174.16 |
| Lorentzian | G | 1581 | 0.68 | 22.01 | 20.68 |
| Lorentzian | Gh | 1590 | 0.27 | 24.68 | 57.20 |
| Lorentzian | D' | 1622 | 0.07 | 2.20 | 20.45 |

| 405 3LS – 2D region | | | | |
|---|---|---|---|---|
| PeakType | Center | Height | Area | FWHM |
| Lorentzian | 2414 | 0.01 | 1.08 | 70.43 |
| Lorentzian | 2550 | 0.01 | 3.61 | 340.23 |
| Lorentzian | 2742 | 0.04 | 4.39 | 68.21 |
| Voigt | 2776 | 0.06 | 5.71 | 62.66 |
| Lorentzian | 2792 | 0.02 | 1.02 | 32.75 |
| Lorentzian (G+D) | 2964 | 0.02 | 4.98 | 165.00 |
| Lorentzian | 3176 | 0.01 | 1.70 | 164.07 |
| Lorentzian | 3246 | 0.01 | 0.27 | 23.07 |

633nm

| | | | |
|---|---|---|---|
| ID/IG | 0.64 | AD/AG | 1.36 |
| ID'/IG | 0.18 | AD'/AG | 0.14 |
| I2D/IG | 0.43 | A2D/AG | 0.99 |
| | | | |
| ID1/IG | 0.03 | AD1/AG | 0.18 |
| ID2/IG | 0.11 | AD2/AG | 0.57 |
| ID3/IG | 0.09 | AD3/AG | 0.60 |

532nm

| | | | |
|---|---|---|---|
| ID/IG | 0.61 | AD/AG | 1.18 |
| ID'/IG | 0.13 | AD'/AG | 0.09 |
| I2D/IG | 0.31 | A2D/AG | 0.73 |
| | | | |
| ID1/IG | 0.04 | AD1/AG | 0.31 |
| ID2/IG | 0.10 | AD2/AG | 0.56 |
| ID3/IG | 0.10 | AD3/AG | 0.46 |

405nm

| | | | |
|---|---|---|---|
| ID/IG | 0.43 | AD/AG | 0.81 |
| ID'/IG | 0.07 | AD'/AG | 0.05 |
| I2D/IG | 0.13 | A2D/AG | 0.24 |
| | | | |
| ID1/IG | 0.01 | AD1/AG | 0.10 |
| ID2/IG | 0.06 | AD2/AG | 0.31 |
| ID3/IG | 0.07 | AD3/AG | 0.25 |

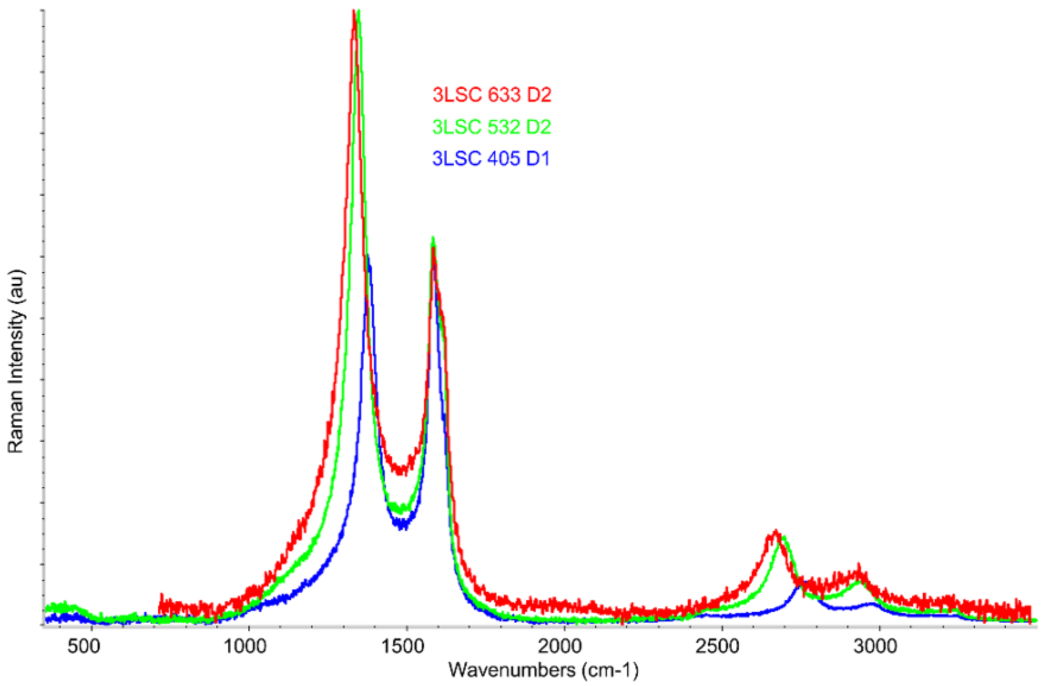


Figure S10(a). Raman spectra of 3L/SC at different exciting laser wavelengths.

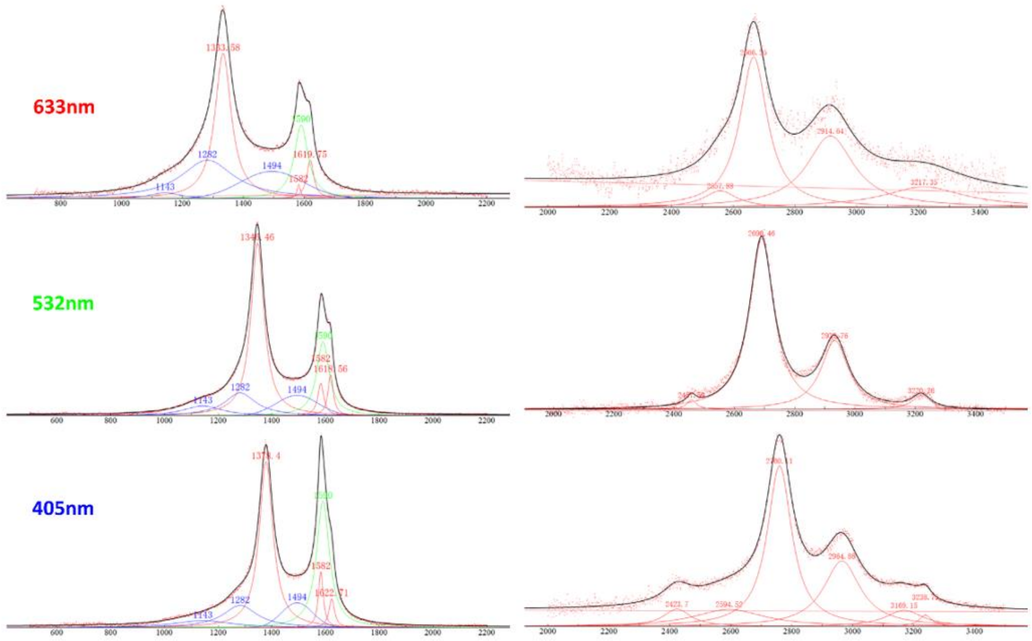


Figure 10(b) Raman spectra of 3L/SC at different excting laser wavelengths: fitting results.

Table S10. Fitting parameters of Raman spectra of 3L/SC at different exciting laser wavelengths and relevant of peaks intensities.

| 633 3LS – first order | | | | | |
|---|---|---|---|---|---|
| PeakType | | Center | Height | Area | FWHM |
| Lorentzian | D1 | 1143 | 0.03 | 5.63 | 117.14 |
| Lorentzian | D2 | 1282 | 0.20 | 65.86 | 211.62 |
| Voigt | D | 1334 | 0.75 | 76.38 | 64.84 |
| Gaussian | D3 | 1494 | 0.14 | 36.34 | 242.73 |
| Lorentzian | G | 1582 | 0.07 | 1.57 | 13.83 |
| Lorentzian | Gh | 1590 | 0.38 | 32.67 | 54.76 |
| Lorentzian | D' | 1620 | 0.20 | 8.65 | 27.72 |

| 633 3LS -2D region | | | | |
|---|---|---|---|---|
| PeakType | Center | Height | Area | FWHM |
| Lorentzian | 2558 | 0.01 | 2.63 | 126.17 |
| Voigt | 2666 | 0.12 | 20.47 | 108.33 |
| Lorentzian (G+D) | 2915 | 0.06 | 16.73 | 186.12 |
| Lorentzian | 3217 | 0.02 | 8.16 | 317.63 |

| 532 3LSC – first order | | | | | |
|---|---|---|---|---|---|
| PeakType | | Center | Height | Area | FWHM |
| Lorentzian | D1 | 1143 | 0.05 | 13.45 | 170.28 |
| Lorentzian | D2 | 1282 | 0.12 | 25.77 | 139.65 |
| Voigt | D | 1346 | 0.89 | 83.39 | 59.62 |
| Gaussian | D3 | 1494 | 0.11 | 20.37 | 182.24 |
| Lorentzian | G | 1582 | 0.17 | 7.08 | 27.08 |
| Lorentzian | Gh | 1590 | 0.38 | 33.62 | 55.97 |
| Lorentzian | D' | 1619 | 0.21 | 7.86 | 23.70 |

| 532 3LSC – 2D region | | | | |
|---|---|---|---|---|
| PeakType | Center | Height | Area | FWHM |
| Lorentzian | 2458 | 0.01 | 0.38 | 38.24 |
| Voigt | 2690 | 0.12 | 18.25 | 98.10 |
| Lorentzian (G+D) | 2933 | 0.05 | 8.18 | 109.51 |
| Lorentzian | 3220 | 0.01 | 1.11 | 73.97 |

| 405 3LSC – first order | | | | | |
|---|---|---|---|---|---|
| PeakType | | Center | Height | Area | FWHM |
| Lorentzian | D1 | 1143 | 0.04 | 14.95 | 251.09 |
| Lorentzian | D2 | 1309 | 0.14 | 39.00 | 173.23 |
| Voigt | D | 1379 | 0.81 | 71.69 | 56.54 |
| Gaussian | D3 | 1494 | 0.13 | 23.20 | 166.64 |
| Lorentzian | G | 1582 | 0.29 | 9.80 | 21.47 |
| Lorentzian | Gh | 1590 | 0.63 | 55.98 | 56.70 |
| Lorentzian | D' | 1622 | 0.15 | 6.10 | 25.43 |

| 405 3LSC – 2D region | | | | |
|---|---|---|---|---|
| PeakType | Center | Height | Area | FWHM |
| Lorentzian | 2424 | 0.01 | 1.79 | 110.508 |
| Lorentzian | 2595 | 0.01 | 4.47 | 280.255 |
| Voigt | 2760 | 0.10 | 14.70 | 97.6642 |
| Lorentzian (G+D) | 2965 | 0.04 | 8.96 | 145.974 |
| Lorentzian | 3169 | 0.01 | 2.25 | 148.331 |
| Lorentzian | 3239 | 0.01 | 0.55 | 52.1083 |

[illegible]

| | | | |
|---|---|---|---|
| ID/IG | 1.66 | AD/AG | 2.23 |
| ID'/IG | 0.44 | AD'/AG | 0.25 |
| I2D/IG | 0.27 | A2D/AG | 0.60 |
| | | | |
| ID1/IG | 0.07 | AD1/AG | 0.16 |
| ID2/IG | 0.44 | AD2/AG | 1.92 |
| ID3/IG | 0.31 | AD3/AG | 1.06 |

532nm

| | | | |
|---|---|---|---|
| ID/IG | 1.62 | AD/AG | 2.05 |
| ID'/IG | 0.38 | AD'/AG | 0.19 |
| I2D/IG | 0.22 | A2D/AG | 0.45 |
| | | | |
| ID1/IG | 0.09 | AD1/AG | 0.33 |
| ID2/IG | 0.21 | AD2/AG | 0.63 |
| ID3/IG | 0.19 | AD3/AG | 0.50 |

405nm

| | | | |
|---|---|---|---|
| ID/IG | 0.88 | AD/AG | 1.09 |
| ID'/IG | 0.17 | AD'/AG | 0.09 |
| I2D/IG | 0.10 | A2D/AG | 0.22 |
| | | | |
| ID1/IG | 0.04 | AD1/AG | 0.23 |
| ID2/IG | 0.16 | AD2/AG | 0.59 |
| ID3/IG | 0.14 | AD3/AG | 0.35 |

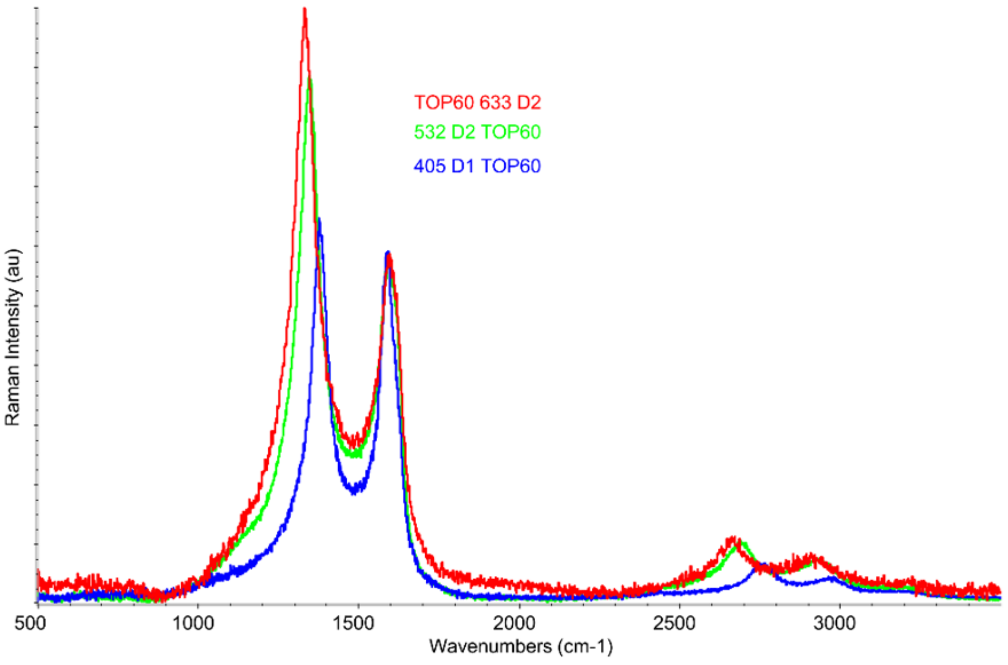


Figure S11(a). Raman spectra of TOP60 at different exciting laser wavelengths.

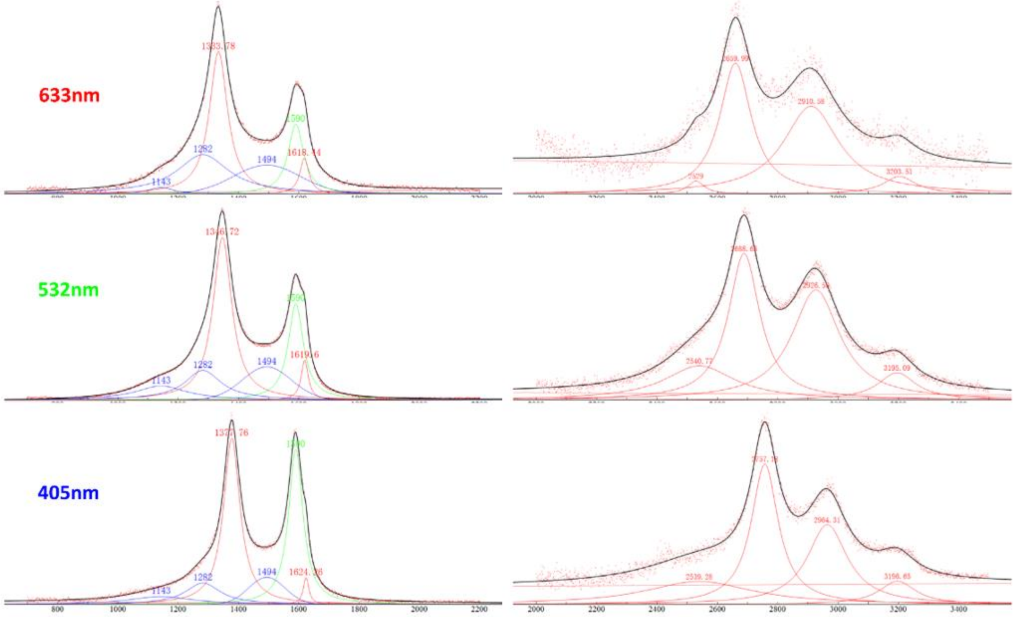


Figure S11(b) Raman spectra of TOP 60 at different exciting laswer wavelengths: fitting results.

Table S11. Fitting parameters of Raman spectra of TOP60 at different exciting laser wavelengths and relevant ratios of peaks intensities.

| 633 TOP60 – first order | | | | | |
|---|---|---|---|---|---|
| PeakType | | Center | Height | Area | FWHM |
| Lorentzian | D1 | 1143 | 0.03 | 5.00 | 99.63 |
| Lorentzian | D2 | 1282 | 0.20 | 66.69 | 208.65 |
| Voigt | D | 1334 | 0.74 | 85.08 | 73.19 |
| Gaussian | D3 | 1494 | 0.15 | 41.58 | 260.81 |
| Lorentzian | G | 1590 | 0.36 | 35.42 | 62.20 |
| Lorentzian | D' | 1618 | 0.19 | 10.55 | 36.31 |

| 633 TOP60 – 2D region | | | | |
|---|---|---|---|---|
| PeakType | Center | Height | Area | FWHM |
| Lorentzian | 2529 | 0.01 | 0.75 | 63.63 |
| Voigt | 2660 | 0.08 | 15.26 | 127.14 |
| Lorentzian (G+D) | 2911 | 0.05 | 17.95 | 223.24 |
| Lorentzian | 3204 | 0.01 | 2.06 | 128.60 |

| 532 TOP60 - first order | | | | | |
|---|---|---|---|---|---|
| PeakType | | Center | Height | Area | FWHM |
| Lorentzian | D1 | 1143 | 0.07 | 22.90 | 201.89 |
| Lorentzian | D2 | 1282 | 0.15 | 35.21 | 146.23 |
| Voigt | D | 1347 | 0.85 | 100.15 | 75.44 |
| Gaussian | D3 | 1494 | 0.17 | 34.83 | 190.45 |
| Lorentzian | G | 1590 | 0.50 | 48.03 | 61.01 |
| Lorentzian | D' | 1620 | 0.21 | 9.23 | 28.56 |

| 532 TOP60 – 2D region | | | | |
|---|---|---|---|---|
| PeakType | Center | Height | Area | FWHM |
| Lorentzian | 2541 | 0.02 | 9.28 | 284.65 |
| Voigt | 2689 | 0.09 | 17.77 | 126.64 |
| Lorentzian (G+D) | 2927 | 0.07 | 20.37 | 193.23 |
| Lorentzian | 3195 | 0.02 | 3.95 | 151.49 |

| 405 TOP60 – first order | | | | | |
|---|---|---|---|---|---|
| PeakType | | Center | Height | Area | FWHM |
| Lorentzian | D1 | 1143 | 0.04 | 16.46 | 255.09 |
| Lorentzian | D2 | 1282 | 0.11 | 24.69 | 141.39 |
| Voigt | D | 1378 | 0.87 | 90.57 | 66.56 |
| Gaussian | D3 | 1494 | 0.14 | 21.24 | 141.29 |
| Lorentzian | G | 1590 | 0.81 | 73.67 | 58.09 |
| Lorentzian | D' | 1624 | 0.14 | 4.86 | 22.15 |

| 405 TOP60 – 2D region | | | | |
|---|---|---|---|---|
| PeakType | Center | Height | Area | FWHM |
| Lorentzian | 2539 | 0.01 | 8.56 | 432.70 |
| Voigt | 2757 | 0.08 | 13.15 | 108.94 |
| Lorentzian (G+D) | 2964 | 0.04 | 11.07 | 161.43 |
| Lorentzian | 3197 | 0.01 | 2.92 | 146.87 |

633nm

| | | | |
|---|---|---|---|
| ID/IG | 2.04 | AD/AG | 2.40 |
| ID'/IG | 0.51 | AD'/AG | 0.30 |
| I2D/IG | 0.21 | A2D/AG | 0.43 |
| | | | |
| ID1/IG | 0.09 | AD1/AG | 0.14 |
| ID2/IG | 0.56 | AD2/AG | 1.88 |
| ID3/IG | 0.41 | AD3/AG | 1.17 |

532nm

| | | | |
|---|---|---|---|
| ID/IG | 1.69 | AD/AG | 2.09 |
| ID'/IG | 0.41 | AD'/AG | 0.19 |
| I2D/IG | 0.18 | A2D/AG | 0.37 |
| | | | |
| ID1/IG | 0.14 | AD1/AG | 0.48 |
| ID2/IG | 0.31 | AD2/AG | 0.73 |
| ID3/IG | 0.34 | AD3/AG | 0.73 |

405nm

| | | | |
|---|---|---|---|
| ID/IG | 1.07 | AD/AG | 1.23 |
| ID'/IG | 0.17 | AD'/AG | 0.07 |
| I2D/IG | 0.10 | A2D/AG | 0.18 |
| | | | |
| ID1/IG | 0.05 | AD1/AG | 0.22 |
| ID2/IG | 0.14 | AD2/AG | 0.34 |
| ID3/IG | 0.17 | AD3/AG | 0.29 |

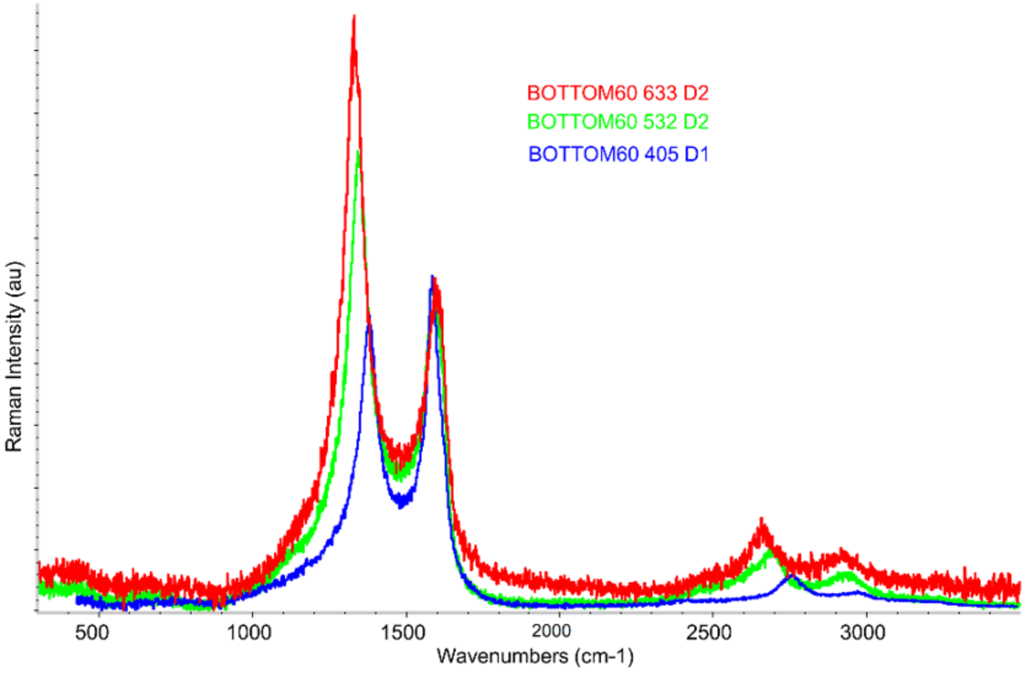


Figure S12 (a) Raman spectra of BOTTOM60 at different exciting laser wavelengths.

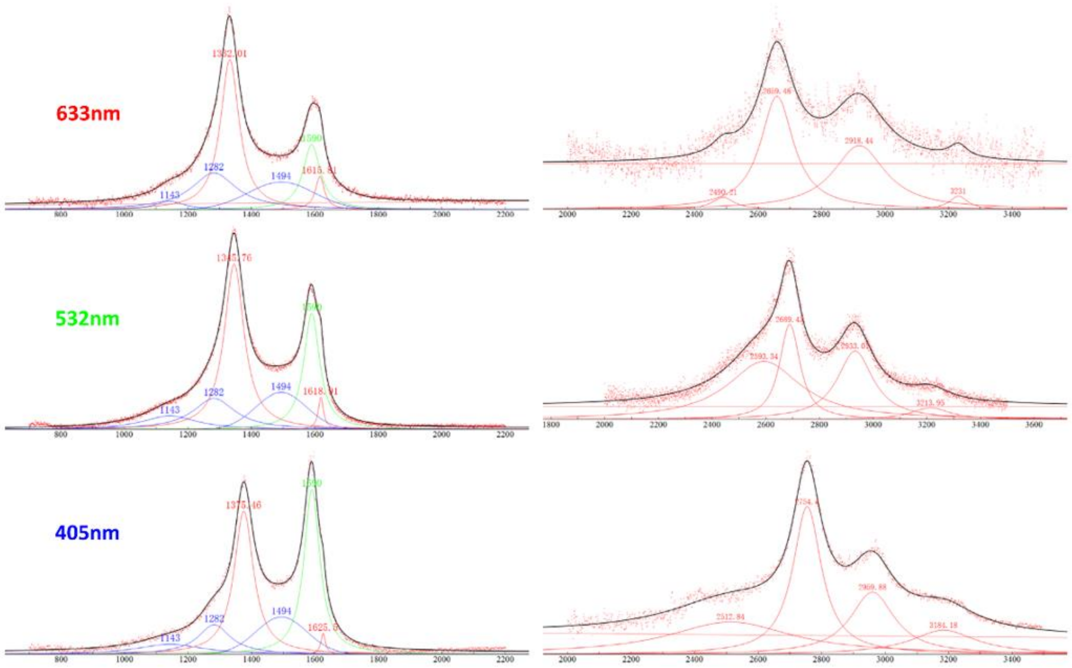


Figure S12 (b). Raman spectra of BOTTOM60 at different exciting laser wavelengths: fitting results.

Table S12. Fitting parametes of Raman spectra of BOTTOM60 at different exciting laser waelengths and relevant ratios of peaks intensities.

| 633 BOTTOM60 – first order | | | | | |
|---|---|---|---|---|---|
| PeakType | | Center | Height | Area | FWHM |
| Lorentzian | D1 | 1143 | 0.04 | 6.93 | 108.30 |
| Lorentzian | D2 | 1282 | 0.18 | 48.83 | 172.68 |
| Voigt | D | 1332 | 0.74 | 82.85 | 71.42 |
| Gaussian | D3 | 1494 | 0.14 | 35.40 | 245.87 |
| Lorentzian | G | 1590 | 0.32 | 33.48 | 66.45 |
| Lorentzian | D' | 1616 | 0.16 | 8.73 | 33.97 |
| **532 BOTTOM60 – first order** | | | | | |
| PeakType | | Center | Height | Area | FWHM |
| Lorentzian | D1 | 1143 | 0.07 | 21.78 | 207.51 |
| Lorentzian | D2 | 1282 | 0.15 | 38.54 | 160.36 |
| Voigt | D | 1346 | 0.82 | 97.15 | 75.72 |
| Gaussian | D3 | 1494 | 0.18 | 36.34 | 187.23 |
| Lorentzian | G | 1590 | 0.57 | 54.58 | 60.57 |
| Lorentzian | D' | 1619 | 0.16 | 4.95 | 19.90 |
| **405 BOTTOM60 – first order** | | | | | |
| PeakType | | Center | Height | Area | FWHM |
| Lorentzian | D1 | 1143 | 0.05 | 19.35 | 247.91 |
| Lorentzian | D2 | 1282 | 0.15 | 28.50 | 124.43 |
| Voigt | D | 1375 | 0.71 | 82.36 | 74.17 |
| Gaussian | D3 | 1494 | 0.18 | 37.08 | 189.25 |
| Lorentzian | G | 1590 | 0.81 | 77.27 | 60.37 |
| Lorentzian | D' | 1626 | 0.10 | 2.80 | 17.22 |

| 633 BOTTOM60 -2D region | | | | |
|---|---|---|---|---|
| PeakType | Center | Height | Area | FWHM |
| Lorentzian | 2490 | 0.01 | 1.21 | 82.09 |
| Voigt | 2659 | 0.09 | 17.43 | 126.35 |
| Lorentzian (G+D) | 2918 | 0.05 | 15.54 | 199.92 |
| Lorentzian | 3231 | 0.01 | 1.13 | 69.79 |
| **532 BOTTOM60 – 2D region** | | | | |
| PeakType | Center | Height | Area | FWHM |
| Lorentzian | 2593 | 0.05 | 24.43 | 330.78 |
| Voigt | 2689 | 0.08 | 10.81 | 91.99 |
| Lorentzian (G+D) | 2933 | 0.06 | 14.14 | 162.94 |
| Lorentzian | 3214 | 0.01 | 2.57 | 171.69 |
| **405 BOTTOM60 – 2D region** | | | | |
| PeakType | Center | Height | Area | FWHM |
| Lorentzian | 2513 | 0.02 | 12.96 | 475.08 |
| Voigt | 2754 | 0.08 | 13.66 | 109.59 |
| Lorentzian (G+D) | 2960 | 0.03 | 8.85 | 168.32 |
| Lorentzian | 3184 | 0.01 | 5.10 | 251.16 |

633nm

| | | | |
|---|---|---|---|
| ID/IG | 2.30 | AD/AG | 2.47 |
| ID'/IG | 0.51 | AD'/AG | 0.26 |
| I2D/IG | 0.27 | A2D/AG | 0.52 |
| | | | |
| ID1/IG | 0.13 | AD1/AG | 0.21 |
| ID2/IG | 0.56 | AD2/AG | 1.46 |
| ID3/IG | 0.42 | AD3/AG | 1.06 |

532nm

| | | | |
|---|---|---|---|
| ID/IG | 1.42 | AD/AG | 1.78 |
| ID'/IG | 0.28 | AD'/AG | 0.09 |
| I2D/IG | 0.13 | A2D/AG | 0.20 |
| | | | |
| ID1/IG | 0.12 | AD1/AG | 0.40 |
| ID2/IG | 0.27 | AD2/AG | 0.71 |
| ID3/IG | 0.32 | AD3/AG | 0.67 |

405nm

| | | | |
|---|---|---|---|
| ID/IG | 0.87 | AD/AG | 1.07 |
| ID'/IG | 0.13 | AD'/AG | 0.04 |
| I2D/IG | 0.10 | A2D/AG | 0.18 |
| | | | |
| ID1/IG | 0.06 | AD1/AG | 0.25 |
| ID2/IG | 0.18 | AD2/AG | 0.37 |
| ID3/IG | 0.23 | AD3/AG | 0.48 |

Table S13-Summary of the parametes obtained by means of curve fitting of the Raman spectra of GNPs and related materials, probed at different exciting laser waelengths.

| Exciting wavelength (nm) - Sample | Peak position in cm$^{-1}$ | | | | | | | Intensity ratio | | | | | | Area ratio | | | | | | | |
|---|---|---|---|---|---|---|---|---|---|---|---|---|---|---|---|---|---|---|---|---|---|
| | D | G | $G_h$ | D' | D1 | D2 | D3 | ID/IG | ID'/IG | I2D/IG | ID1/IG | ID2/IG | ID3/IG | AD/AG | AD'/AG | A2D/AG | AD1/AG | AD2/AG | AD3/AG | FWHM (G) | ID/ID' |
| 633-HOPG | - | 1582 | - | - | - | - | - | - | - | 0.53 | - | - | - | - | - | 1.5 | - | - | - | 14.29 | - |
| 633-Rod Graphite (1) | 1336 | 1583 | - | 1621 | - | 1282 | 1494 | 0.16 | 0.06 | 0.66 | - | 0.01 | 0.01 | 0.38 | 0.05 | 1.35 | - | 0.02 | 0.01 | 16.82 | 2.67 |
| 633-Rod Graphite (2) | 1334 | 1580 | - | 1617 | 1143 | 1282 | 1494 | 0.45 | 0.11 | 0.61 | 0.02 | 0.04 | 0.02 | 0.72 | 0.08 | 1.24 | 0.08 | 0.14 | 0.02 | 24.22 | 4.09 |
| 633-3L | 1336 | 1582 | - | 1619 | - | - | - | 0.21 | 0.05 | 0.51 | - | - | - | 0.49 | 0.07 | 0.89 | - | - | - | 19.09 | 4.20 |
| 633-3L/S | 1336 | 1582 | 1590 | 1621 | 1143 | 1282 | 1494 | 0.64 | 0.18 | 0.43 | 0.03 | 0.11 | 0.09 | 1.36 | 0.14 | 0.99 | 0.18 | 0.57 | 0.60 | 19.00 | 3.56 |
| 633-3L/SC | 1334 | 1582 | 1590 | 1620 | 1143 | 1282 | 1494 | 1.66 | 0.44 | 0.27 | 0.07 | 0.44 | 0.31 | 2.23 | 0.25 | 0.6 | 0.16 | 1.92 | 1.06 | 54.76 | 3.77 |
| 633-TOP60 | 1334 | - | 1590 | 1618 | 1143 | 1282 | 1494 | 2.04 | 0.51 | 0.21 | 0.09 | 0.56 | 0.41 | 2.4 | 0.30 | 0.43 | 0.14 | 1.88 | 1.17 | 62.20 | 4.00 |
| 633-BOTTOM60 | 1332 | - | 1590 | 1616 | 1143 | 1282 | 1494 | 2.3 | 0.51 | 0.27 | 0.13 | 0.56 | 0.42 | 2.47 | 0.26 | 0.52 | 0.21 | 1.46 | 1.06 | 66.45 | 4.51 |
| | | | | | | | | | | | | | | | | | | | | | |
| 532-HOPG | - | 1582 | - | - | - | - | - | - | - | 0.44 | - | - | - | - | - | 1.19 | - | - | - | 13.18 | - |
| 532-Rod Graphite (1) | 1351 | 1583 | - | 1624 | - | 1282 | 1494 | 0.14 | 0.04 | 0.58 | - | 0.00 | 0.00 | 0.28 | 0.02 | 1.17 | - | 0.09 | 0.01 | 17.54 | 3.50 |
| 532-Rod Graphite (2) | 1350 | 1581 | | 1622 | - | 1282 | - | 0.21 | 0.05 | 0.5 | - | 0.01 | - | 0.37 | 0.03 | 1.11 | - | 0.11 | - | 20.40 | 4.20 |
| 532-3L | 1350 | 1581 | - | 1622 | - | - | - | 0.17 | 0.03 | 0.52 | - | - | - | 0.36 | 0.02 | 0.95 | - | - | - | 18.65 | 5.67 |
| 532-3L/S | 1349 | 1581 | 1590 | 1622 | 1143 | 1282 | 1494 | 0.61 | 0.13 | 0.31 | 0.04 | 0.10 | 0.10 | 1.18 | 0.09 | 0.73 | 0.31 | 0.56 | 0.46 | 18.95 | 4.69 |
| 532-3L/SC | 1346 | 1582 | 1590 | 1619 | 1143 | 1282 | 1494 | 1.62 | 0.38 | 0.22 | 0.09 | 0.21 | 0.19 | 2.05 | 0.19 | 0.45 | 0.33 | 0.63 | 0.50 | 55.97 | 4.26 |
| 532-TOP60 | 1347 | - | 1590 | 1620 | 1143 | 1282 | 1494 | 1.69 | 0.41 | 0.18 | 0.14 | 0.31 | 0.34 | 2.09 | 0.19 | 0.37 | 0.48 | 0.73 | 0.73 | 61.01 | 4.12 |
| 532-BOTTOM60 | 1346 | - | 1590 | 1619 | 1143 | 1282 | 1494 | 1.42 | 0.28 | 0.13 | 0.12 | 0.27 | 0.32 | 1.78 | 0.09 | 0.2 | 0.40 | 0.71 | 0.67 | 60.57 | 5.07 |
| | | | | | | | | | | | | | | | | | | | | | |
| 405-HOPG | - | 1581 | - | - | - | - | - | - | - | 0.16 | - | - | - | - | - | 0.44 | - | - | - | 14.11 | - |
| 405-Rod Graphite (1) | 1381 | 1581 | - | 1625 | - | 1282 | 1494 | 0.06 | 0.01 | 0.21 | - | 0.00 | 0.00 | 0.14 | 0.00 | 0.39 | - | 0.01 | 0.01 | 17.69 | 6.00 |
| 405-Rod Graphite (2) | 1382 | 1582 | - | 1624 | - | - | - | 0.15 | 0.01 | 0.22 | - | - | - | 0.27 | 0.00 | 0.51 | - | - | - | 22.22 | 15.00 |
| 405-3L | 1381 | 1581 | - | 1622 | - | - | - | 0.06 | 0.01 | 0.25 | - | - | - | 0.12 | 0.00 | 0.54 | - | - | - | 18.75 | 6.00 |
| 405-3L/S | 1379 | 1581 | 1590 | 1622 | 1143 | 1282 | 1494 | 0.43 | 0.07 | 0.13 | 0.01 | 0.06 | 0.07 | 0.81 | 0.05 | 0.24 | 0.10 | 0.31 | 0.25 | 20.68 | 6.14 |
| 405-3L/SC | 1379 | 1582 | 1590 | 1622 | 1143 | 1282 | 1494 | 0.88 | 0.17 | 0.1 | 0.04 | 0.16 | 0.14 | 1.09 | 0.09 | 0.22 | 0.23 | 0.59 | 0.35 | 56.70 | 5.18 |
| 405-TOP60 | 1378 | - | 1590 | 1624 | 1143 | 1282 | 1494 | 1.07 | 0.17 | 0.1 | 0.05 | 0.14 | 0.17 | 1.23 | 0.07 | 0.18 | 0.22 | 0.34 | 0.29 | 58.09 | 6.29 |
| 405-BOTTOM60 | 1375 | - | 1590 | 1626 | 1143 | 1282 | 1494 | 0.87 | 0.13 | 0.1 | 0.06 | 0.18 | 0.23 | 1.07 | 0.04 | 0.18 | 0.25 | 0.37 | 0.48 | 60.37 | 6.69 |

Table S14. (a) Description and number of Loretzian/Voigt components adopted in the fit of the Raman spectra of some selected samples. (b) Curve type selected for the individual components.

**(a)**

| **Samples** | **First order** | **Second order** |
|---|---|---|
| HOPG | 1 (G) | 3 (2D) |
| ROD (i) | 5 (D, G, D’, D2, D3) | 4 (2D), small D+(G,D’) |
| ROD (ii) | 7 (D, G, Gh, D’,D1, D2, D3) | 3 (2D), small D+(G,D’) |
| 3L/P | 3 (D, G, D’) | 4 (2D) |
| 3L/S | 7 (D, G, Gh, D’, D1, D2, D3) | 3 (2D), D+(G,D’) |
| 3L/SC | 7 (D, G, Gh, D’, D1, D2, D3) | 2 (2D), D+(G,D’) |
| TOP60 | 6 (D, Gh, D’, D1, D2, D3) | 2 (2D), D+(G,D’) |
| BOTTOM60 | 6 (D, Gh, D’, D1, D2, D3) | 2 (2D), D+(G,D’) |

**(b)**

| **Fitting components** | **Type of peak** |
|---|---|
| D1 (1143) | Lorentian |
| D2 (1282) | Lorentian |
| D | Voigt |
| D3 (1494) | Gaussian |
| $G,G_h$ | Lorentian |
| D’ | Lorentian |
| 2D | Voigt |
| other two quanta | Lorentian |

The deconvolution of the Raman spectra of TOP60, BOTTOM60, 3L/SC, 3L/S, 3L/P, ROD and HOPG obtained with 633, 532 and 405 nm excitation is illustrated in Figs. S5-S12 and Tables S5-S13, while Table S14 summarizes some general characteristic of the fitting for the spectra anlyzed, namely the number of components necessary for the fitting of the G-D region (first-order Raman spectrum) and for the 2D region for each sample. Table S14 (b) highlights the type of the fitting curves adopted for each component (Lorentzian or Voigt functions). The number of components

required is almost the same for the same sample, irrespective to the exciting wavelength.

The first order Raman spectrum (D and G bands region, red dots in Figures S5-S12) is fitted by the suitable number of single Voigt or Lorentian components which generate the reconstructed spectrum (the black line). The second order (2D) region consists of the structured 2D band (from 2 to 4 components according to the sample) and of additional minor components in (2466 and 3244 $cm^{-1}$ in the spectrum of HOPG ($\lambda_{exc}$= 633 nm) and a G+D component ($\nu$~2920 $cm^{-1}$), which is remarkably important in the case of GNPs. For all the spectra, the relevant parameters of the individual components are provided in Tables S5-S13.

Sadezky et al.[1,2] described a five peak model for the fitting of the first order Raman spectra of carbon soot and related carbonaceous materials, and Claramunt et al.[3] proposed a six peak model for the fitting of Raman data of GO. In the present study, we find that each different sample requires an increasing number of components, according to its complexity. Starting from HOPG, showing the one only G band nicely fitted by a lorentzian curve, ROD graphite requires from 5 to 7 components, according to the point of sampling, thus revaling a inhomogeneous morphology. Seven peaks were necessary to fit the Raman spectra of our GNPs. Interestingly, in the case of 3L samples two different components are required for the fit of the G bands: the lower wavenumber one, corresponding to the “graphite-like” peak at 1582 $cm^{-1}$ and a higher wavenumber one (1590 $cm^{-1}$) which corresponds to the one only G component found for TOP60 and BOTTOM60 nanoparticles.

The peak frequency of the bands included in the fitting, have been obtained in the following way:

(i) Three major components correspond to the main G, D, D’ lines. The frequency of D and D’ were initially set to the corresponding band maxima of each original spectrum and refined during the fitting. The center of G line is kept fixed (see spectra of HOPG, ROD and 3L/P). In the cases of 3L/S and 3L/SC samples the G band has a structured shape, and it is fitted by a G

a Gh peak. The frequency of Gh was set at the corresponding band maximum value of the original spectrum of the TOP60 and/or BOTTOM60. For the samples of TOP60, BOTTOM60, the G peak disappears completely and is replaced by Gh.

(ii) In several cases, to obtain a good fit it is necessary to add three further components, which allow describing a region on the lower frequency side of the D band (D1 and D2 components) and in a region between the D and the G bands (D3 component). A preliminary fit with adjustable peaks frequency values showed that a common set of peak frequencies (namely 1143, 1282 and 1494 $cm^{-1}$) can be adopted – and kept fixed - for all our samples at any exciting wavelength.

(iii) For TOP60, BOTTOM60 and 3L/SC samples, the 2D peaks can be well fitted by a single Voigt peak. However, for ROD or 3L/P samples, we need multiple Lorentian peaks and a Voigt peak to carefully fit the 2D band, due to the contribution of graphite-like phases showing multiple layer stacking. According to different preparation processes, different samples will show a D+G feature at different wavenumber, which is fitted by a Lorentian.

*GNP Raman Spectra evolution upon the photons flux*

As the Figure S13 shown, in the case of BOTTOM60, remarkable and irreversible change of the D band, that weakens while increasing the power of the laser beam from 0.5 mW to 5 mW. However, hardly any shifts occur on TOP60 samples.

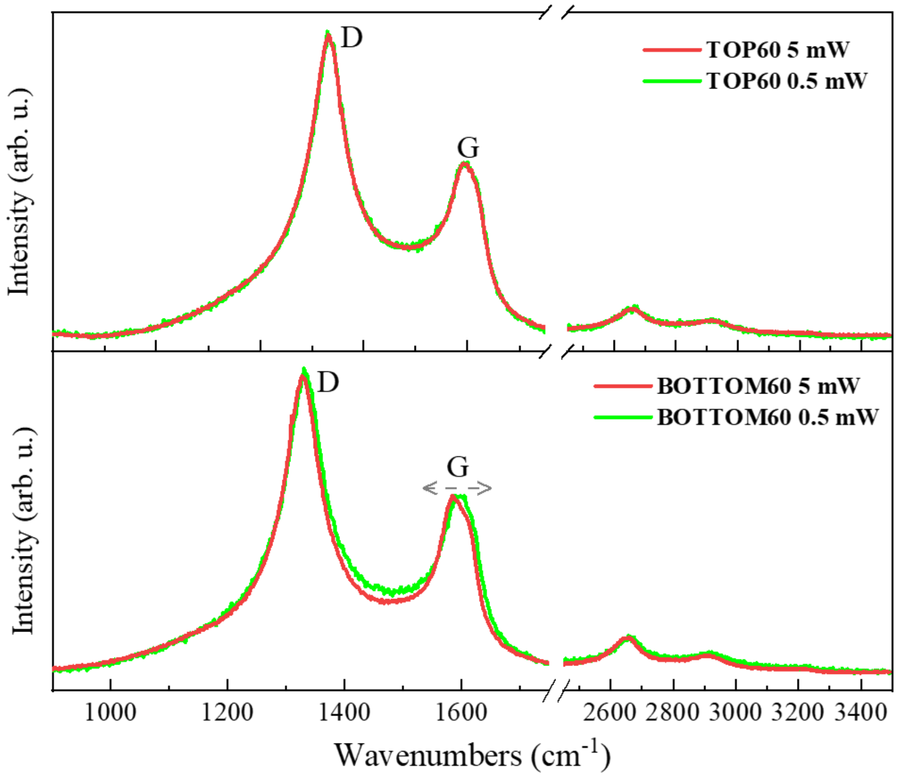


Figure S13. Raman spectra of the GNPs samples of TOP60 and BOTTOM60 excited by red (633 nm) laser at the different power values, in the same point.

*Multiple Raman sampling and GNPs homogeneity*

Raman spectra of GNPs were collected at different locations on the same sample with different wavelength of laser, as shown in Figure S14. For TOP60 NPs, the Raman spectra at different locations look very similar both with 633 nm and 532 nm laser excitation. The Raman spectra of BOTTOM60 at different locations are similar, while probing with the 633 nm laser. However, under laser excitation at 532 nm, the Raman spectra of BOTTOM60 show differences when recorded at different locations. This suggests that the BOTTOM60 sample is less homogeneous than TOP60 and/or more sensitive to the weak changes of the power of laser.

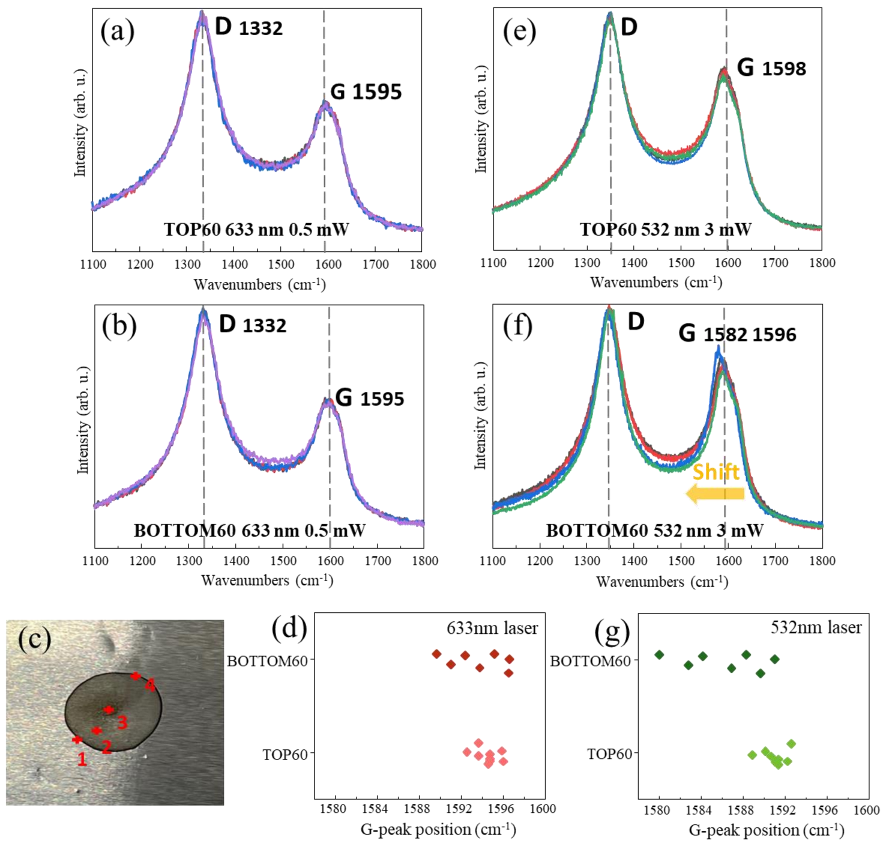


Figure S14. Raman spectra of the GNP samples of TOP60, BOTTOM60 excited by different laser wavelength. (a,b) the D and G band region with 633 nm laser excitation; (c) sampling points on GNPs tiled on an aluminum substrate; (e,f) the D and G band region with 532 nm laser excitation; and (d) and (g) changes of G-peak position.

## References


[1]. Sadezky, A.; Muckenhuber, H.; Grothe, H.; Niessner, R.; Pöschl, U. Raman Microspectroscopy of Soot and Related Carbonaceous Materials: Spectral Analysis and Structural Information. *Carbon* **2005**, *43*, 1731–1742.

[2]. Ivleva, N.; McKeon, U.; Niessner, R.; Pöschl, U. Raman Microspectroscopic Analysis of Size-Resolved Atmospheric Aerosol Particle Samples Collected with an Elpi: Soot, Humic-Like Substances, and Inorganic Compounds. *Aerosol Sci. Technol.* **2007**, *41*, 655–671.

[3]. Claramunt, S.; Varea, A.; Lopez-Diaz, D.; Velázquez, M.; Cornet, A.; Cirera, A. The importance of interbands on the interpretation of the Raman spectrum of graphene oxide. *J. Phys. Chem. C.* **2015**, *119*, 10123–10129.
